# Photodynamic therapy: a promising alternative for high-grade squamous intraepithelial lesion treatment

**DOI:** 10.3389/fonc.2026.1899399

**Published:** 2026-08-07

**Authors:** Lara Termini, Cristina Paula Castanheira, Noely Paula Cristina Lorenzi, Enrique Boccardo, Telma Lisboa-Nascimento, Maurício S. Baptista, Natalia Mayumi Inada, Waleska Kerllen Martins

**Affiliations:** 1 Center for Translational Research in Oncology, Instituto do Cancer do Estado de Sao Paulo ICESP, Hospital das Clinicas da Faculdade de Medicina da Universidade de Sao Paulo FMUSP HC, São Paulo, Brazil; 2 Comprehensive Center for Precision Oncology, Universidade de Sao Paulo, São Paulo, Brazil; 3 Department of Obstetrics and Gynecology, Hospital das Clinicas da Faculdade de Medicina da Universidade de Sao Paulo FMUSP HC, São Paulo, Brazil; 4 Department of Gynecology, Hospital Universitario da Universidade de Sao Paulo, São Paulo, Brazil; 5 Department of Microbiology, Instituto de Ciências Biomédicas, Universidade de Sao Paulo, São Paulo, Brazil; 6 Bioengineering Graduate Program, University of Brazil, São Paulo, Brazil; 7 Department of Biochemistry, Institute of Chemistry, University of Sao Paulo, São Paulo, Brazil; 8 Institute of Physics, University of Sao Paulo, São Carlos, Brazil; 9 Lumo Life Science - Instituto de Ciência, Tecnologia e Inovação, São Paulo, Brazil

**Keywords:** 5-aminolevulinic acid (5-ALA), autophagy restoration, cervical intraepithelial neoplasia (CIN), high-risk human papillomavirus (hrHPV), hrHPV clearance, precision oncology, protoporphyrin IX, pyroptosis

## Abstract

Cervical cancer, driven by persistent infection with high-risk human papillomavirus (hrHPV), remains a major global health burden. While conventional excisional procedures such as loop electrosurgical excision (LEEP) and conization are effective in treating high-grade squamous intraepithelial lesions (HSIL), which include cervical intraepithelial neoplasia grades 2 and 3 (CIN2 and CIN3, respectively), they are associated with significant risks to cervical integrity and adverse obstetric outcomes. Photodynamic therapy (PDT), particularly using the protoporphyrin IX precursors 5-aminolevulinic acid (5-ALA) and its derivatives, hexyl aminolevulinate (HAL), and methyl aminolevulinate (MAL), has emerged as an alternative and minimally invasive strategy to treat younger women with CIN2. Here, we delineate the cellular and molecular mechanisms that underlie PDT’s efficacy in the treatment of cervical lesions. We emphasize the ability of 5-ALA-PDT to selectively target dysplastic cells while preserving the anatomical and functional integrity of the cervix. Central to this therapeutic effect is the modulation of cell death pathways. Specifically, we discuss how PDT counteracts the inhibitory effects of hrHPV on autophagy, thereby facilitating viral clearance and restoring homeostatic immune responses. Furthermore, we delineate the multifaceted cell death modalities orchestrated by 5-ALA-PDT, underscoring their pivotal role in the targeted eradication of hrHPV-transformed cervical cells. Clinical data supporting the reduction of viral load and high regression rates of HSIL are summarized, alongside a critical evaluation of current treatment protocols. Finally, we propose that the future of cervical PDT lies in precision oncology. This highlights the need to focus on the identification of resistance biomarkers and the development of therapeutic strategies that exploit the synergistic combination of PDT with molecular modulators to optimize clinical outcomes and prevent recurrence.

## Highlights

- Molecular selectivity: Use of 5-ALA precursors allows for the selective accumulation of protoporphyrin IX in dysplastic cells, enabling targeted cytotoxic effects.
- Viral clearance via autophagy: 5-ALA-PDT counteracts the hrHPV-mediated inhibition of autophagy, promoting the degradation of viral proteins and enhancing the host immune response.
- Integrated phototoxicity: 5-ALA-PDT orchestrates a multifaceted landscape of cell death—synergizing apoptosis, ferroptosis, pyroptosis, necroptosis and immunogenic cell death, while simultaneously restoring autophagic flux to circumvent hrHPV-mediated immune evasion and facilitate viral clearance.
- Future synergy: Optimizing efficacy requires a shift toward combination therapies that target specific molecular resistance determinants and autophagic flux.
- Organ preservation: 5-ALA-PDT offers a superior safety profile compared to LEEP/conization by preserving cervical function and reducing reproductive complications.

## 1 Introduction

Cervical cancer is a significant global health challenge, ranking eighth in overall cancer incidence globally (1). Symptoms may include abnormal vaginal bleeding, pelvic pain, and pain during sexual intercourse (2). Almost the totality of cervical squamous cell carcinomas is associated with persistent infection by high-risk human papillomavirus (hrHPV) (3).

Despite the potential for primary prevention through HPV vaccination, improved outcomes through early diagnosis, and treatment, cervical cancer represents 3.3% of all human cancers and 6.8% of all cancers in females (1). Besides, in this latter group, this disease accounts for 8.1% of all cancer-associated deaths, emerging as the fourth leading cause of cancer-related mortality among women worldwide (1). Even though the global adoption of HPV vaccines increases, with over 83% (162/194 countries) of World Health Organization (WHO) member states incorporating them into national immunization programs and 63% of girls completing the planned vaccination (4); it is estimated that nearly 67% of women between 20 and 70 years of age worldwide have never undergone HPV testing (5). Consequently, cervical cancer will persist as a major global public health challenge, particularly in regions with limited access to screening and vaccination programs.

This underscores the fact that hrHPV-associated tumors and precursor lesions will continue to be diagnosed in the next decades, reinforcing the need for effective therapeutic interventions. Consequently, early detection and treatment of intraepithelial cervical lesions are critical to preventing progression into invasive cervical squamous cell carcinoma. Photodynamic therapy (PDT) has emerged as a promising alternative for the treatment of pre-cancerous lesions, offering comparable therapeutic efficacy with fewer side effects and superior preservation of cervical function (6–8).

To advance the therapeutic potential of PDT in hrHPV-associated disease, future research must integrate mechanistic, translational, and clinical perspectives. In this review, beyond addressing the photophysical parameters and clinical applications of PDT, we provide a state-of-the-art overview of the cellular and molecular modulatory mechanisms mediated by PDT, the principal molecular pathways targeted by hrHPV, and the defining hallmarks of invasive cervical cancer.

## 2 Cervical cancer

**Figure 1:**
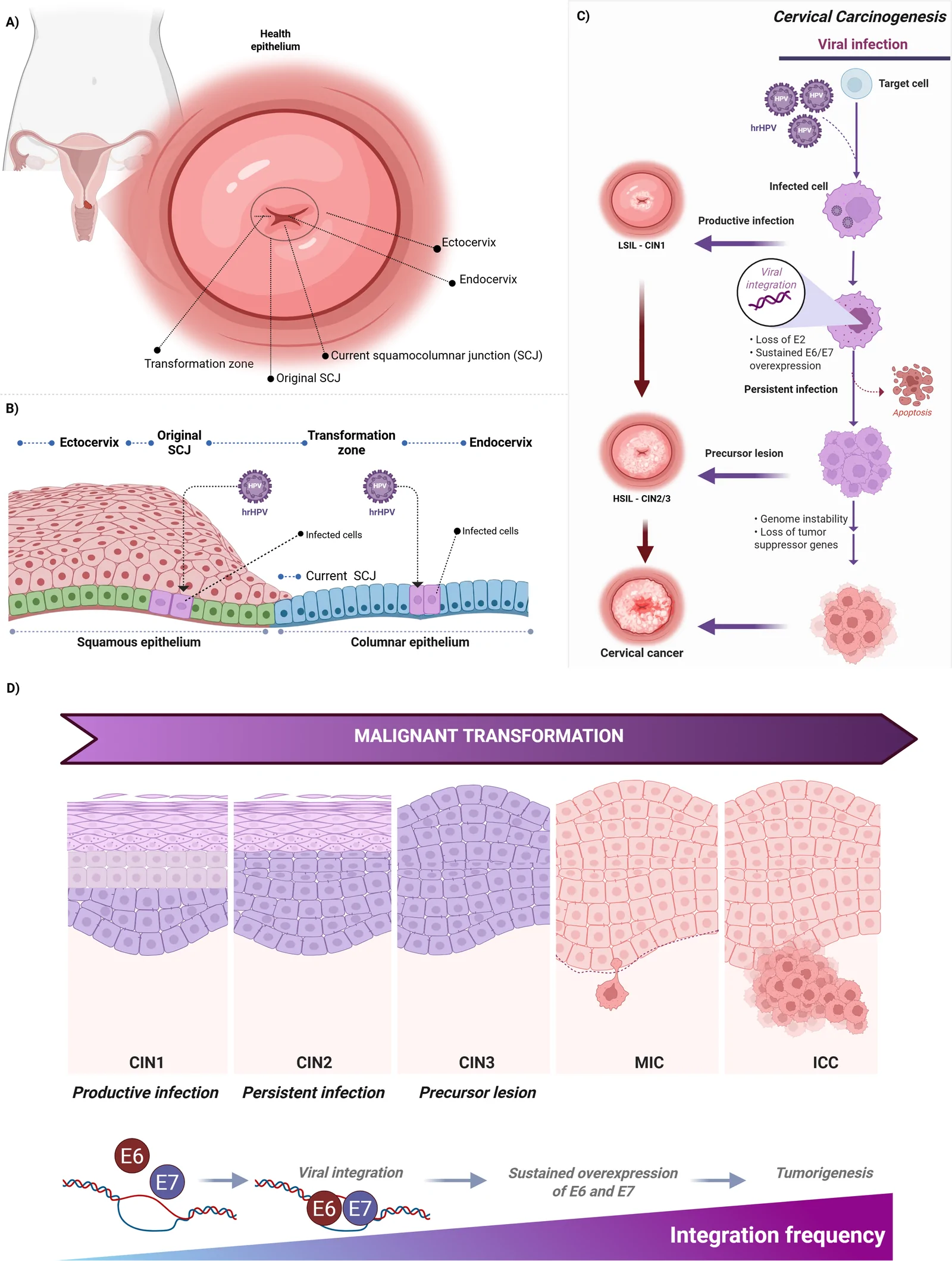
Anatomical and histopathological progression of hrHPV-associated cervical lesions during cervical carcinogenesis. **(A)** The cervix is composed of the ectocervix (stratified squamous epithelium) and the endocervix (simple columnar epithelium). The transformation zone, located between the original and the current SCJ, is a dynamic region of squamous metaplasia and is particularly susceptible to persistent hrHPV infection. Consequently, it represents the principal site for the development of hrHPV-associated cervical precancer and cancer. **(B)** Cellular targets of hrHPV infection: High-risk HPV genotypes, particularly HPV16 and HPV18, infect basal epithelial cells exposed by epithelial microtrauma and may also target reserve cells and specialized SCJ cells within the transformation zone. This anatomically and biologically distinct region provides a permissive environment for persistent hrHPV infection, making it the principal site for the development of cervical intraepithelial neoplasia and cervical cancer. **(C)** Molecular pathogenesis of hrHPV-mediated cervical carcinogenesis: Cervical carcinogenesis is a multistep process initiated by persistent infection with high-risk HPV. Most infections are transient and resolve spontaneously; however, persistent infection may give rise to productive low-grade squamous intraepithelial lesions (LSIL/CIN1). Continued deregulation of the viral oncogenes E6 and E7 promotes genomic instability, functional inactivation of the tumor suppressor proteins p53 and pRb, and progression to high-grade squamous intraepithelial lesions (HSIL/CIN2–3). In a subset of cases, viral DNA integration into the host genome further enhances oncogene deregulation and contributes to malignant progression. Without appropriate treatment, such as excisional procedures, a proportion of HSIL lesions may ultimately progress to invasive cervical carcinoma. **(D)** Chronological molecular pathogenesis of hrHPV-mediated cervical carcinogenesis: Schematic representation of the continuum of cervical disease from initial persistent high-risk HPV infection through progressive epithelial transformation: hrHPV infection → LSIL/CIN1 → HSIL/CIN2 → HSIL/CIN3 → MIC → ICC. Transitional vectors illustrate the progressive deregulation of viral E6/E7 oncogene expression, increasing genomic instability, accumulation of host genetic and epigenetic alterations, and, in a subset of lesions, viral DNA integration during progression toward invasive disease. CIN, cervical intraepithelial neoplasia; ICC, invasive cervical carcinoma; hrHPV, high-risk human papillomavirus; LSIL, low-grade squamous intraepithelial lesion; HSIL, high-grade squamous intraepithelial lesion; MIC, microinvasive carcinoma; SCJ, squamocolumnar junction. Created in https://BioRender.com.

Cervical cancer (CC) is a consequence of persistent hrHPV infection, evolving through a well-characterized path: from low-grade squamous intraepithelial lesions (LSIL) to high-grade squamous intraepithelial lesions (HSIL), and ultimately microinvasive carcinoma (MIC) and CC itself (**Figure 1**). LSIL corresponds to cervical intraepithelial neoplasia (CIN) with mild dysplasia (CIN1), while HSIL includes moderate dysplasia (CIN2) and severe dysplasia (CIN3) (9, 10). While CIN1 lesions often regress spontaneously, CIN2/3 lesions carry a significant risk of progression to MIC or invasive cervical cancer (ICC). In fact, CIN3 represents carcinoma *in situ* and timely intervention can significantly reduce the risk of developing invasive CC (11).

### 2.1 Etiology

Infection with a sub-group of HPV types from the alpha-genus that exhibit tropism for mucosal epithelia is the etiologic agent in the development of genital warts and cervical intraepithelial neoplasias. Mucosal HPV types are transmitted predominantly through sexual contact. More than 450 types of HPV have been fully sequenced. Among these, 17 types (HPV16, 18, 26, 31, 33, 35, 39, 45, 51, 52, 56, 58, 59, 68, 69, 73, 82) exhibit carcinogenic potential and are classified as hrHPV types due to their association with high-grade lesions and CC. In addition, 11 HPV types (HPV6, 11, 40, 42, 43, 44, 54, 61, 70, 81, and CP6108) are classified as low-risk and are primarily associated with benign conditions such as genital warts. Among these, HPV6 and HPV11 account for more than 90% of genital wart cases (12–14).

HPV16 and HPV18 are the most prevalent types, collectively accounting for approximately 71% of CC cases globally (13). Besides, HPV31, 33, 45, 52 and 58 types cause nearly an additional 20% of ICC cases, contributing significantly to the global burden of this disease (15). Over 90% of HPV infections resolve spontaneously within 6–18 months. However, persistent infection represents a critical determinant in the development of high-grade squamous intraepithelial lesion (CIN2/3) and cervical cancer (16–18). Persistent hrHPV infections drive abnormal cellular proliferation, inhibit apoptosis, and induce genomic instability, thereby facilitating the accumulation of genetic alterations that underlie malignant progression (19–21).

There are national routine immunization schedules based on commercially available vaccines, bivalent (HPV16 and 18), quadrivalent (HPV6, 11, 16, and 18), and nonavalent (HPV6, 11, 16, 18, 31, 33, 45, 52, and 58), to prevent hrHPV infection and CC development. Considering the public health perspective, “*the bivalent, quadrivalent, and nonavalent vaccines offer comparable immunogenicity, efficacy, and effectiveness for the prevention of cervical cancer, which is mainly caused by HPV types 16 and 18*” (World Health Organization (WHO, 2022).

Persistent hrHPV infection is the most critical factor for the development of CC precursor lesions and their malignant progression. However, it is not sufficient on its own. Host intrinsic factors such as the number of sexual partners, age, immune response, presence of mutations, smoking, stress, long-term use of oral contraceptives, multiparity, microbiome, presence of other sexually transmitted infections, multicentric, multifocal, and persistent hrHPV infection, as well as factors of the virus itself (like viral type, variant, viral load, frequency and location of integration), influence the probability of the emergence of invasive lesions (22). The interplay between hrHPV and CC is complex, involving various molecular mechanisms that lead to the transformation of normal cells into cancerous ones. Understanding these mechanisms is crucial for developing targeted therapies and improving prevention strategies against CC.

### 2.2 The histological gateway to hrHPV infection

The uterine cervix serves as a complex histological gateway, where the structural transition between distinct epithelial lineages dictates the localization of oncogenic events. As detailed in **Figure 1**, the cervical epithelium is structurally complex, comprising three principal regions: Ectocervix: characterized by stratified squamous epithelium, with a basal layer expressing p63, cytokeratin 5 (KRT5), and cytokeratin 14 (KRT14)—markers of epithelial stratification and mechanical integrity (23, 24); Transformation Zone (TZ): the dynamic interface between squamous and columnar epithelia, where reserve cells reside beneath the columnar layer (23, 25). Reserve cells near the squamous-columnar junction (distal reserve cells) strongly express KRT5 and cytokeratin 17 (KRT17), which are markers of squamous differentiation, meanwhile cytokeratin 7 (KRT7) and cytokeratin 8 (KRT8) are associated with columnar epithelium (24, 26). Thus, these cells can differentiate into both squamous and columnar lineages (27), and Endocervix: lined by a single layer of columnar cells, with less distinct basal cells but containing reserve cells capable of multipotent differentiation (23).

The basal layer of the cervical epithelium is regionally specialized and heterogeneous. Only a subset of cells are true progenitors, and their potency and differentiation state depend on their anatomical niche (26). The biological “hotspot” for malignancy, however, lies within the TZ (**Figure 1**). This dynamic interface is populated by multipotent reserve cells that harbor a unique plastic phenotype. It is precisely this cellular plasticity, coupled with the high turnover of the squamous-columnar junction, that renders the TZ exquisitely susceptible to hrHPV infection and the subsequent cascade of dysplastic progression toward invasive carcinoma.

The transition from a persistent hrHPV infection to ICC is not an abrupt event but rather the culmination of a protracted evolutionary process within the cervical microenvironment (28). As detailed in **Figure 1**, it underscores the critical shift from intraepithelial confinement to stromal infiltration. While low-grade lesions frequently undergo spontaneous resolution, the emergence of high-grade squamous intraepithelial lesions (HSIL) represents a definitive tipping point in disease progression (28, 29). As illustrated, the structural breakdown of the basement membrane marks the transition to MIC, eventually leading to ICC. This final stage is characterized by deep stromal invasion and a total loss of epithelial stratification, highlighting the biological necessity of identifying virus presence and critical morphological alterations before the lesion escapes local control.

Given the unique susceptibility of the TZ reserve cells, as illustrated in **Figure 1**, understanding the progression to malignancy requires a deeper interrogation of the viral-host interface. The following section details the canonical and non-canonical mechanisms of hrHPV entry, the specific role of heparan sulfate proteoglycans in viral docking. Besides, we described the signaling pathways affected by E6 and E7 oncoproteins that contribute to establishing a permissive environment for oncogenic transformation.

### 2.3 hrHPV-induced genetic and epigenetic pathways in cervical cancer development

HPV is a small icosahedral non-enveloped virus (50 to 60 nm in diameter), which contains a double-stranded DNA molecule (between 7000 and 8000 bp), functionally divided into three regions: early, late, and the long control region (LCR) or upstream regulatory region (URR) (29). The LCR comprises a non-coding region of HPV that plays a pivotal role in the regulation of viral gene expression and replication. This region contains critical elements such as the origin of replication (Ori), early promoters (P97 in HPV16 and P105 in HPV18), and binding sites for viral proteins (E1 and E2) and host transcription factors, such as C-JUN (AP1), KRF-1, NF1, Oct-1, Sp1, TEF-10, and YY1. The LCR is essential for initiating viral DNA replication and transcription, serving as a hub for the interaction between viral and host factors (29).

The second segment, known as the “early (E) region,” comprises the open reading frames (ORFs) for E1, E2, E4, E5, E6, and E7 genes. The proteins E1 and E2 primarily contribute to viral genome replication and transcription. E4 is involved in virion release. E5 plays an important role by contributing to host proliferation and immune evasion, while E6 and E7 play a primary role in creating a cellular milieu suitable for productive viral replication and persistence (see below). The third segment, referred to as the “late (L) region,” constitutes 40% of the viral genome and includes the ORFs L1 and L2 that encode the major and minor viral capsid proteins, respectively (29). Among the viral proteins encoded by hrHPV types, E1, E2, E6, and E7 play pivotal roles in the viral life cycle and oncogenesis. E1 and E2 are essential for viral replication and genome maintenance. E1 functions as a helicase and origin recognition factor, facilitating the replication of viral DNA. E2 regulates viral transcription and replication by binding to specific sequences in the viral genome, ensuring proper control of viral gene expression and copy number. Disruption of E2 expression, often due to viral genome integration into host chromosomes, leads to deregulated expression of E6 and E7 (20).

The viral oncoproteins E6 and E7 are central to the hrHPV life cycle and malignant transformation. They reprogram differentiated keratinocytes to re−enter the cell cycle, driving them back into S phase to sustain viral genome replication and amplification—a mechanism observed in both oncogenic and non−oncogenic HPV types. E6 from hrHPV types promotes the degradation of the tumor suppressor p53, thereby blocking apoptosis and permitting the accumulation of genetic alterations. It also activates telomerase, contributing to cellular immortalization. In parallel, E7 targets pRb, disrupting its control over E2F transcription factors and enforcing uncontrolled cell cycle progression. Beyond this, E7 induces genomic instability through processes such as centrosome amplification and DNA damage, further accelerating malignant transformation (20). Together, these viral proteins orchestrate the disruption of key cellular pathways, including cell cycle regulation, apoptosis, and genomic stability, ultimately facilitating the development of cervical intraepithelial lesions and their progression to CC. The multiple and growing number of HPV oncoproteins exceed the scope of this review. For a detailed description of these viral proteins activity, the reader is referred to some excellent reviews (20, 30, 31).

Persistent hrHPV infections, primarily by HPV16 and HPV18, lead also to changes such as DNA methylation, histone modifications, and non-coding RNA dysregulation. These changes often silence tumor suppressor genes (e.g., *CADM1, MAL, FAM19A4*, and *miR-124-2*) disrupt cellular processes like apoptosis, cell cycle regulation, and establish oncogenic transcriptional programs. Together, these epigenetic modifications contribute to the progression from hrHPV infection to CC, offering potential biomarkers and therapeutic targets for improved diagnosis and treatment.

These molecular mechanisms provide insights into the pathogenesis of cervical cancer and highlight potential targets for diagnosis, treatment, and prevention strategies. Improved knowledge of hrHPV’s oncogenic mechanisms could lead to the development of novel therapeutic targets and enhance early diagnosis of cervical malignancies (29).

### 2.4 Stemness and dormancy: distinct cellular states in ICC

The progression of CC is marked by the dynamic interplay between cellular stemness and dormancy, two distinct yet interconnected states that significantly influence tumor evolution and therapeutic resistance. Stemness, characterized by self-renewal capacity and multipotency, is a hallmark of cervical cancer stem cells (CCSCs). Dormancy, on the other hand, represents a state of cellular quiescence, where cells temporarily exit the cell cycle, reducing their metabolic activity and proliferation while maintaining the potential to re-enter active growth under favorable conditions.

Cervical epithelial cells with self-renewal capacity properties, such as basal and reserve cells, are considered potential candidates for transformation into cervical malignant cells upon infection by hrHPV types (32). Unlike reserve cells, most basal cells are unipotent, maintaining squamous histology rather than exhibiting multipotency (25, 33).

Under the influence of persistent hrHPV infection, genomic instability, and somatic mutations, stem cell-like cells, such as reserve cells, may transform into CCSCs. This transformation is characterized by a gradual loss of non-malignant stem cell properties (e.g., high proliferation and low differentiation) and an increase in cancer stem cell properties, such as epithelial-mesenchymal transition (EMT), invasion, and metastasis. EMT is a key factor in the malignant transformation and may serve as a crucial characteristic that differentiates CCSCs from cervical stem/progenitor cells (27).

Single-cell transcriptomic analyses have revealed that CCSCs undergo significant molecular and functional changes during the progression from HSIL to invasive cervical squamous carcinoma (27). Epithelial clusters specific to the HSIL stage exhibited an elevated stemness score compared to most other epithelial clusters. However, higher enrichment of cervical stem cell-related gene signatures (CSS) gradually decreased with lesion progression. Low CSS was significantly associated with poor prognosis and lower progression free survival (27). The reserve markers KRT5, KRT17, and p63 were primarily found in HSIL and cervical cancer groups, particularly in malignant epithelial subclusters (33). KRT17 was identified as a malignant cell marker and showed progressively increased expression during the transition from HSIL to CC (33).

The HSIL and MIC stages exhibited an intermediate CSS profile, reflecting a transitional state between the normal and ICC stages. However, p53 and apoptosis were highly expressed in HSIL than in normal or malignant cervical stages, which may render them more responsive to extrinsic modulators of regulated cell death. In addition, HSIL revealed the highest CSS related to normal cervix (e.g., E2F activation, G2/M checkpoint, PI3K/AKT/mTOR signaling, DNA repair, and proliferation), as well as CSS linked to invasion, metastasis and hypoxia (27).

Overexpression of YAP (Yes-associated protein) and TAZ (transcriptional co-activator with PDZ-binding motif) enhances the stemness of HSIL cells, as evidenced by increased sphere formation and upregulation of cancer stem cell markers (CD44, CD133, KLF4, Sox2) (27). Notably, YAP/TAZ activation promotes glycolysis, increasing glucose consumption, lactic acid production, and extracellular acidification rate. This metabolic reprogramming supports the cervical malignant transformation and their proliferation, invasion, and migration (34).

OCT4, SOX2, and NANOG are key transcription factors associated with the regulation of pluripotency and self-renewal CCSCs (27). Their elevated levels are indicative of enhanced stemness, which contributes to the CCSCs’ ability to self-renew, resist apoptosis, and drive tumor progression, recurrence, and metastasis (35).

In non-malignant epithelial stem cells (NESCs), CSS was predominantly characterized by enrichment of the gene signatures related to inflammation, cell cycle regulation (G2/M checkpoint control), DNA damage response, DNA repair pathways, MYC and E2F target activation, fatty acid metabolism, and oxidative phosphorylation. By contrast, CSS in the ICC stage displayed downregulation of tumor suppressor-related signatures (e.g., G2/M checkpoint control) and strong enrichment of invasion, metastasis, EMT, angiogenesis, IL-6 signaling, Wnt and TGF-β pathways, glycolysis, and hypoxia signatures (27).

The enriched energy metabolism signatures suggested an enhanced glycolysis signature (Warburg effect) in CCSCs at ICC stage and a stronger oxidative phosphorylation (OXPHOS) signature in NESCs (27). In HSIL and MIC, OXPHOS remains a prominent metabolic pathway, which exhibits transitional features between NESCs and CSCs. OXPHOS activity reflects the high mitochondrial content, and energy demands of these cells, supporting their proliferative capacity and low differentiation state. However, as the disease progresses toward ICC, a metabolic shift occurs, with reduced OXPHOS activity and increased reliance on Warburg effect, even in the presence of oxygen. This metabolic reprogramming supports rapid cell division and survival in hypoxic tumor microenvironments (36). This shift reflects a loss of non-malignant stem cell properties and the emergence of tumor-specific stemness, enabling CCSCs to adapt to the tumor microenvironment and evade therapeutic interventions (27).

Dormant cervical cancer cells (DCCCs), in contrast, represent a quiescent subpopulation within the tumor microenvironment (27). These cells are characterized by upregulation of dormancy-associated pathways, including SNAI2-mediated G0/G1 cell cycle arrest, reduced glucose metabolism, and suppression of proliferative signaling (37). Dormancy provides a survival advantage, allowing DCCCs to resist conventional therapies and contribute to tumor recurrence and metastasis (38). Interestingly, while CCSCs are key drivers of tumor initiation and progression, DCCCs play a critical role in maintaining long-term tumor persistence and therapeutic resistance (39, 40).

Therapeutic strategies targeting these distinct cellular states must account for their unique vulnerabilities. For instance, CCSCs in HSIL and MIC, with their high mitochondrial activity and glycolytic metabolism, may be particularly susceptible to mitochondrial-targeted therapies such as 5-aminolevulinic acid-based photodynamic therapy. In contrast, DCCCs, with their reduced metabolic activity and quiescent state, may require approaches that disrupt dormancy-associated survival pathways. Autophagy inhibitors like chloroquine, hydroxychloroquine, and ULK1/AMPK inhibitors (e.g., SBI-0206965) can block the lysosome-dependent degradation pathway that sustains dormant cells. Ferroptosis inducers such as erastin and RSL3 trigger lipid peroxidation-dependent cell death, targeting the antioxidant defenses of dormant cells. Additionally, redox disruptors like NRF2 inhibitors amplify ROS toxicity, while oxidative phosphorylation and fatty acid oxidation inhibitors block energy production, destabilizing dormant cells. These approaches aim to eliminate dormant cells and prevent tumor recurrence (38).

In conclusion, the dual states of stemness and dormancy in ICC represent critical determinants of tumor progression, therapeutic resistance, and recurrence. Understanding the molecular mechanisms underlying these states and their transitions offers valuable insights into the development of targeted therapies aimed at eradicating both proliferative and dormant cancer cell populations. By addressing the unique vulnerabilities of CCSCs and DCCCs, it may be possible to improve treatment outcomes and reduce the burden of cervical cancer. In the next section, we will discuss the common therapeutic strategies elicit to treat intraepithelial cervical lesions.

## 3 Treatment strategies

Treatment of cervical intraepithelial lesions seeks to prevent progression of invasive cancer while avoiding unnecessary intervention. Management is individualized according to lesion severity, visibility of the transformation zone, patient age, reproductive plans, and risk estimates derived from testing.

Excisional procedures like loop electrosurgical excision procedure (LEEP), large loop excision of the transformation zone (LLETZ), cold knife conization, or laser cone biopsy are preferred for histologic HSIL (CIN3 or persistent CIN2). Ablative techniques including cryotherapy and laser CO_2_ ablation are acceptable when the lesion is fully visualized, confined to the ectocervix, and does not extend into the endocervical canal. In practice, excisional treatment is more commonly favored in some settings because it provides a histologic specimen (2). However, these treatments may result in cervical damage and carry a heightened risk of various complications, including an increased risk of premature birth, postoperative bleeding, infection, cervical stenosis, and spontaneous abortion in future pregnancies.

In case of low-grade squamous intraepithelial lesions and for selected cases of CIN2 when fertility preservation is a priority, active surveillance is preferred. Surveillance combines periodic colposcopy with hrHPV-based testing. CIN2 shows frequent spontaneous regression, particularly in younger patients, supporting conservative management when appropriate (2).

For nonpregnant patients aged 25 years and older with a calculated immediate risk of CIN3 (≥60%) or worse at or above commonly accepted thresholds, excisional treatment without prior biopsy confirmation is preferred (2). However, colposcopy with biopsy remains an acceptable alternative. Decisions should be made through shared decision-making that weighs cancer prevention against potential reproductive consequences.

Despite not yet being widely incorporated into guideline recommendations such as the 2019 ASCCP Risk-Based Management Consensus Guidelines (2), PDT has emerged as a promising, fertility-sparing option for the treatment of cervical intraepithelial neoplasias in selected women (41).

**Figure 2:**
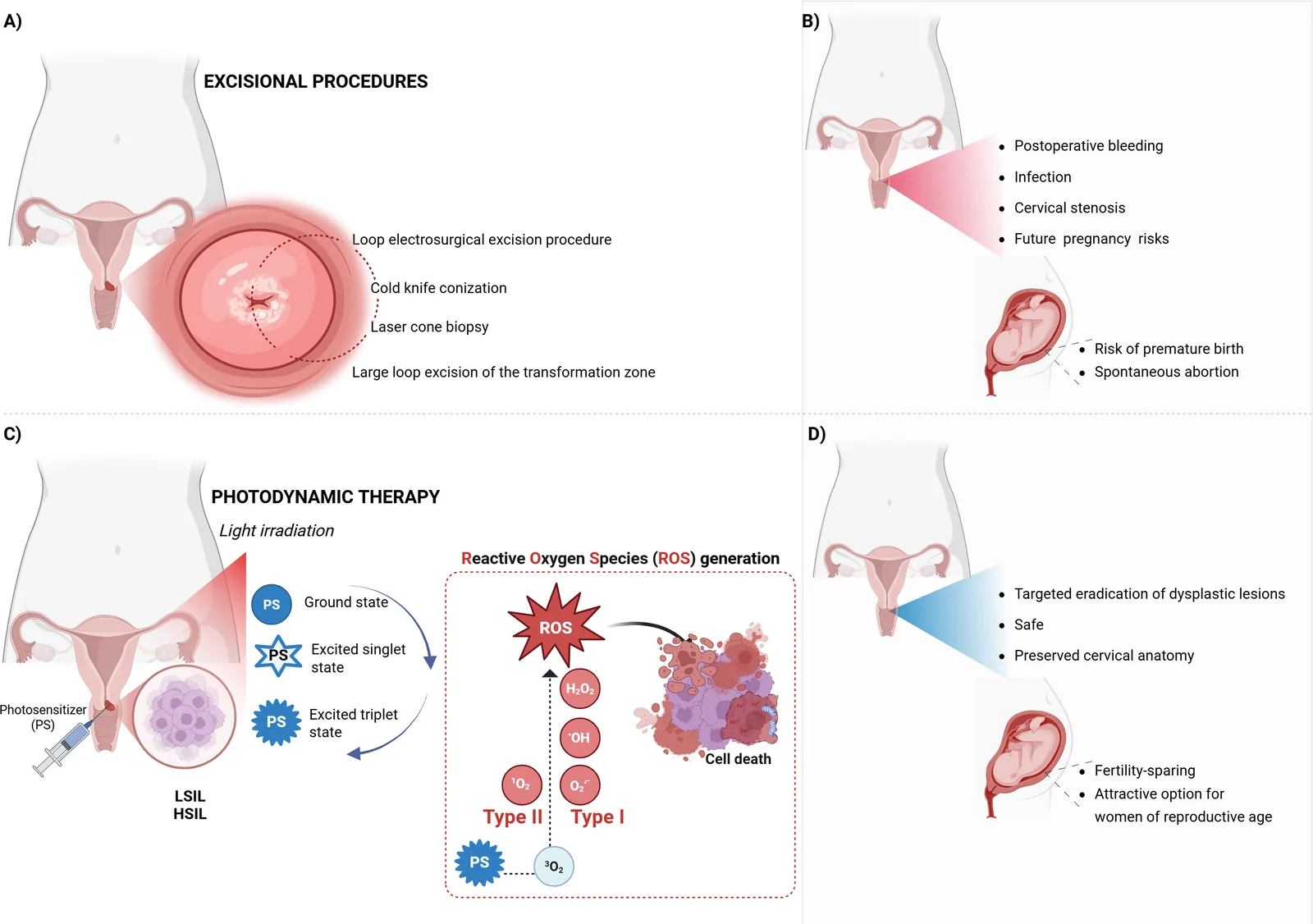
Integrated clinical management and photodynamic mechanisms in the treatment of cervical intraepithelial lesions. **(A)** Conventional excisional modalities. Schematic representation of surgical interventions for the removal of the cervical transformation zone, including LEEP, cold knife conization, laser cone biopsy, and LLETZ. These methods rely on the physical removal of dysplastic tissue to prevent progression to invasive carcinoma. **(B)** Clinical complications of surgical intervention. While effective, excisional procedures are associated with significant morbidity. Acute risks include postoperative bleeding and infection, while long-term anatomical sequelae such as cervical stenosis may occur. Critically, the reduction in cervical structural integrity poses substantial future pregnancy risks, including increased incidences of spontaneous abortion and premature birth. **(C)** Mechanism of action of PDT. PDT offers a non-invasive, targeted alternative for treating LSIL and HSIL. Upon administration, the photosensitizer (PS) selectively accumulates in dysplastic cells. Subsequent light irradiation at a specific wavelength promotes the PS from its ground state to an excited singlet state, followed by intersystem crossing to a long-lived excited triplet state. The triplet state PS interacts with molecular oxygen (^3^O_2_) via two pathways: Type I reactions (electron transfer) producing radicals such as superoxide anion (O_2_^•-^), hydroxyl (^•^OH), and hydrogen peroxide (H_2_O_2_); and Type II reactions (energy transfer) producing highly reactive singlet oxygen (^1^O_2_). The resulting burst of ROS induces localized oxidative stress, leading to selective cell death. **(D)** Clinical advantages and fertility preservation. Unlike excisional methods, PDT allows for the targeted eradication of dysplastic lesions while preserving cervical anatomy. This approach is characterized by high safety profiles and a lack of adverse obstetric outcomes, establishing PDT as a highly attractive, fertility-sparing option for women of reproductive age seeking to maintain cervical function. HSIL, high-grade squamous intraepithelial lesion; LEEP, loop electrosurgical excision procedure; LLETZ, large loop excision of the transformation zone; LSIL, low-grade squamous intraepithelial lesion; PDT, Photodynamic Therapy, PS, photosensitizer; ROS, reactive oxygen species. Created in https://BioRender.com.

The PDT mechanism involves a dynamic oxidative reaction in the presence of molecular oxygen (^3^O_2_), a photosensitizing chemical agent (PS), and light at a specific wavelength (visible spectrum), which aligns with the absorption spectrum of the PS. The photophysical principles stemming from photosensitization in PDT are based on the absorption of electromagnetic energy at a specific wavelength by the PS in presence ^3^O_2_ (**Figure 2**).

The clinical efficacy and selectivity of PDT are determined less by the photochemical efficiency of the PS and more by its precise subcellular localization. A central paradigm emerging is that subcellular distribution overrides quantum yield as the primary determinant of biological outcome. Membrane-bound photosensitizers, for instance, permeabilize organelles orders of magnitude more efficiently than broadly distributed counterparts. A consequence of the direct PS-lipid double bond contact, which favors Type I electron-transfer reactions over singlet oxygen oxidation. This contact-dependent mechanism generates truncated lipids and initiates radical chain propagation, amplifying oxidative damage far beyond what the initial reactive oxygen species (ROS) alone could achieve. Consequently, targeting specific organelles yields distinct therapeutic outcomes: mitochondrial photodamage triggers regulated cell death, while lysosomal targeting induces cathepsin release and autophagy dysfunction, often proving more effective for long-term cell killing. Furthermore, the amplification inherent light-induced peroxidation processes explain why even modest light doses can overwhelm cellular antioxidant defenses, shifting from homeostasis (eustress) to oxidative distress (42).

The PS can be administered intravenously, intraperitoneally, or topically to the patient, with the tumor region being selectively irradiated. Although these components (PS and light) are harmless individually, when combined, they provide localized and effective antitumor therapy, which does not harm healthy cells, resulting in minimal side effects. The combination of PS and light leads to the generation of reactive excited states (singlet and triplet excited states), as well as various reactive oxygen species (ROS), such as singlet oxygen (^1^O_2_), hydroxyl radical (^•^OH), superoxide anion (O_2_^•-^), and hydrogen peroxide (H_2_O_2_). All these reactive species can efficiently oxidize and irreversibly damage target tumor tissues/cells (43). Although PDT can preserve cervical anatomy and reproductive potential compared with excisional approaches, definitive conclusions require larger, randomized studies with longer follow-up to confirm durability of response, oncologic equivalence, and effects on fertility. Among all photosensitizers investigated for the management of cervical intraepithelial lesions (e.g., chlorin e6, phthalocyanines, methylene blue, and others), porphyrin derivatives or precursors highlight as the most investigated agents (41).

Thus, PDT represents a minimally invasive, fertility-sparing alternative that enables targeted eradication of dysplastic lesions with reduced collateral tissue injury, making it a particularly attractive option for women of reproductive age. The following section reviews the photochemical, photobiological aspects of PDT based on porphyrin precursors, which can drive neoplasias into regulated death.

## 4 Porphyrin-mediated PDT in cervical neoplasia

In contrast to other PSs used for PDT-mediated cell death, porphyrin precursors offer distinct clinical advantages, as we discussed here. They enable porphyrin accumulation in dysplastic and neoplastic cells, offering tissue-selective cytotoxicity while preserving cervical architecture and reproductive potential. First-generation PSs, most notably Photofrin^®^ (porfimer sodium/dihematoporphyrin ether) administered either topically or intravenously, achieved one-year complete remission (CR) rates of 72.7% to 96.1% across CIN grades 1–3 following irradiation with 630 nm light at energy densities of 100–140 J/cm² (44–46). However, the relapse occurrences 3.9-27.3% and the incidence of prolonged cutaneous photosensitivity in 22.6–48% of patients limited its widespread adoption (44–46).

Photofrin^®^ achieved hrHPV eradication in 72–74% of patients with CIN3 at three months post-treatment. The eradicated types included HPV16, 18, 31, 35, 51, 52, 58, 61, 70, 82, and others. Nevertheless, 22% of CIN3 cases remained HPVDNA positive at 12 months after Photofrin^®^-mediated PDT (45). hrHPV typing frequently changed post-treatment, suggesting possible reinfection with different viral types (45, 46). Importantly, persistent or recurrent hrHPV infection did not correlate with disease recurrence, as patients with hrHPV persistence or re-infection exhibited no CIN relapse during follow-up (46).

The advent of second-generation PSs, particularly δ-aminolevulinate or 5-aminolevulinic acid (5-ALA), has addressed these limitations through enhanced selectivity for dysplastic epithelium, topical administration, and a markedly improved safety profile, thereby establishing a new standard for PDT in gynecologic oncology. 5-ALA is a highly hydrophilic molecule, as demonstrated by its negative log P value (approximately -1.5) and high aqueous solubility (see **Supplementary Table**).

The scientific foundation for 5-ALA-PDT was established in the 1990s through seminal work by Pottier and Kennedy, who elucidated its underlying mechanisms and highlighted its clinical potential (47, 48). Early clinical trials confirmed this feasibility and safety for CIN treatment, demonstrating that epithelial tissue exhibited markedly higher fluorescence compared to the underlying stroma (49). Subsequent meta-analyses have validated its efficacy compared to control and tolerability (50).

Clinical evidence demonstrates that 5-ALA–mediated PDT achieves high complete remission (CR) and hrHPV clearance rates across CIN1–3, with outcomes ranging from 84.5% to 90.9% CR and 64.4% to 92.5%hrHPV clearance at 6–12 months post-treatment. Despite these encouraging results, a proportion of patients still present with persistent lesions (7.1–25%) or recurrence (~2.3%), underscoring that therapeutic efficacy is not uniformly sustained (49, 51–59).

These relapse rates highlight the need to move beyond clinical endpoints and interrogate the molecular and biochemical determinants of treatment response. A deeper understanding of these pathways could identify predictive biomarkers of relapses and inform strategies to optimize PDT efficacy. Ultimately, integrating molecular insights with clinical data is essential to reduce recurrence and enhance durable remission in patients undergoing 5-ALA–PDT. In the next section we discuss how the pharmacokinetic and localization properties make 5-ALA particularly well suited for fertility-sparing, mucosal applications, allowing spatially confined phototoxic effects and supporting fluorescence-guided diagnosis and therapy.

### 4.1 5-ALA metabolism and heme biosynthesis: foundations for selective photodynamic therapy in cervical lesions

**Figure 3:**
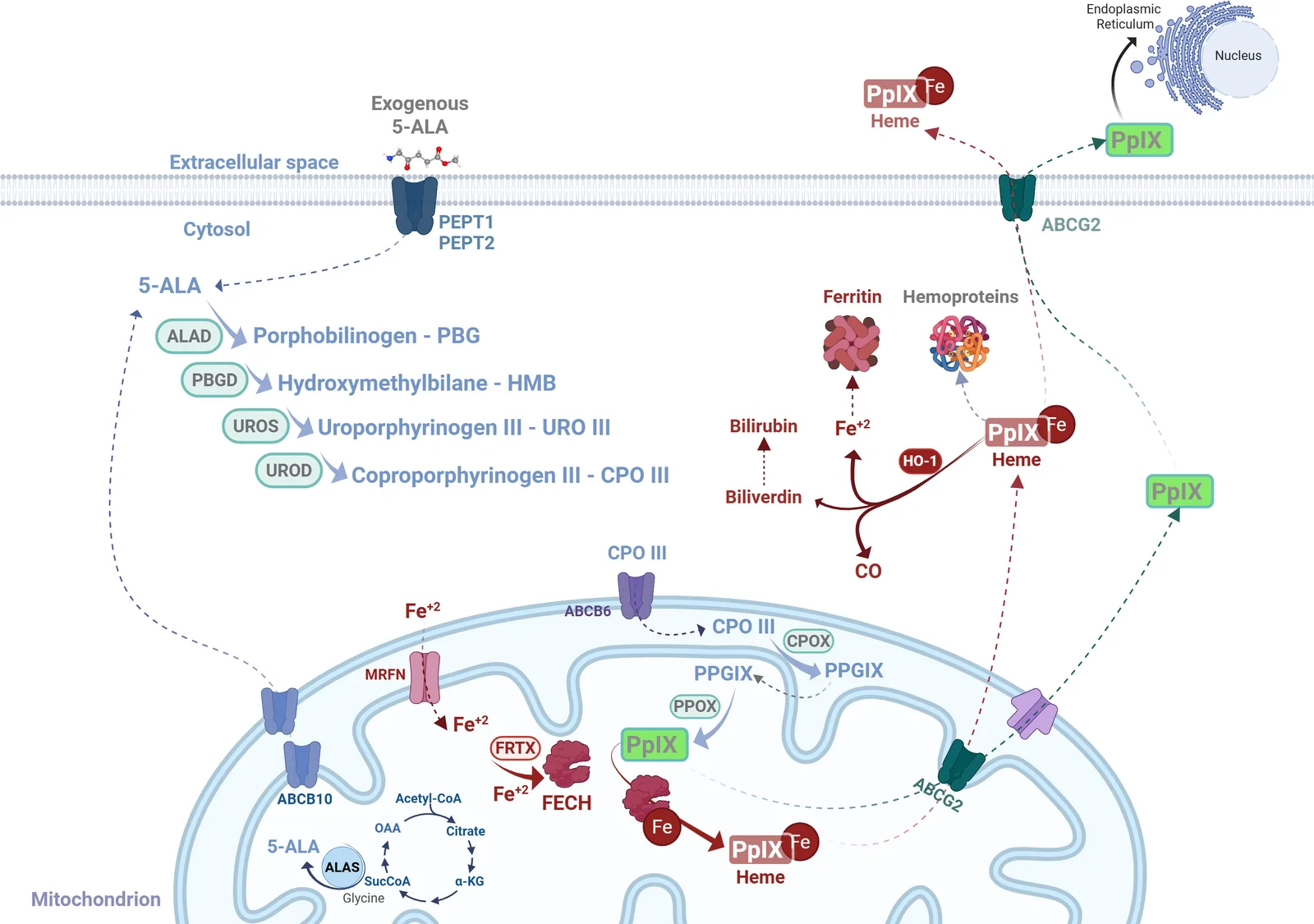
Metabolic regulation of the heme biosynthetic pathway and protoporphyrin IX (PpIX) accumulation. Schematic representation of the intracellular conversion of exogenous 5-ALA into the potent photosensitizer PpIX. Exogenous uptake and cytosolic cascade: Exogenous 5-ALA is internalized via the peptide transporters PEPT1 and PEPT2. Once in the cytosol, 5-ALA undergoes a series of enzymatic transformations: dehydratase (ALAD) converts 5-ALA into PBG, which is further processed by PBGD to form HMB. UROS converts HMB into URO III, which is then decarboxylated by UROD to form CPO III. Mitochondrial processing and PpIX synthesis: CPO III is translocated into mitochondria via the ATP-binding cassette ABCB6 transporter. Within the mitochondrial intermembrane space and matrix, CPO III is oxidized to PPGIX by CPOX, and subsequently to PpIX by PPOX. In normal physiological conditions, PpIX is converted into heme by FECH through the insertion of ferrous iron (Fe^2+^), a process regulated by FRTX and mitochondrial iron importers (MRFN). Metabolic fate and efflux: Heme serves as a prosthetic group for various hemoproteins or is degraded by HO-1 into biliverdin, CO, and Fe^2+^. Intracellular levels of PpIX and heme are further modulated by the efflux transporter ABCG2. Once in the extracellular space, PpIX can be taken up by other organelles, such as endoplasmic reticulum. Iron ions can be stored by ferritin, while biliverdin is converted into bilirubin by BVR. ABCG2, ATP-binding cassette subfamily G member 2; 5-ALA, 5-aminolevulinic acid; ALAD, aminolevulinic acid dehydratase; BVR, biliverdin reductase; CO, carbon monoxide; CPO III, coproporphyrinogen III; CPOX, coproporphyrinogen oxidase; FECH, ferrochelatase; FRTX, frataxin; HMB, hydroxymethylbilane; HO-1, heme oxygenase-1; MRFN, mitoferrin; PBG, porphobilinogen; PBGD, porphobilinogen deaminase; PPGIX, protoporphyrinogen IX; PpIX, Protoporphyrin IX; UROS, uroporphyrinogen III synthase; UROD, uroporphyrinogen decarboxylase; CPO III, coproporphyrinogen III; PpIX, protoporphyrin IX; PPGIX, protoporphyrinogen IX. Created in https://BioRender.com.

5-ALA (5-aminolevulinic acid) belongs to the class of organic compounds, known as δ-amino acids and their derivatives (8). It occurs naturally as precursor in the porphyrin/heme biosynthetic pathway (**Figure 3**). Therefore, 5-ALA-PDT exhibits fewer toxic effects compared to Photofrin^®^, as it is metabolized into protoporphyrin IX (PpIX), which undergoes selective photoactivation within cervical epithelium (49). This metabolic property renders 5-ALA a safer and more targeted PS for PDT in the treatment of CIN.

Endogenously, 5-ALA is synthesized within the mitochondrial matrix, functioning as the initial precursor in the heme biosynthetic Pathway (**Figure 3**). In contrast, exogenous administration of 5-ALA circumvents the enzymatic step mediated by ALA synthase (ALAS), thereby markedly accelerating the downstream accumulation of PpIX (8). When administered exogenously 5-ALA can be transported across the plasma membrane peptide transporter 1 and 2 (PEPT1 and PEPT2) (60, 61)—**Figure 3**.

Through a series of enzymatic reactions in the cytosol, 5-ALA is metabolized into coproporphyrinogen III (CPO III). Subsequently, protoporphyrinogen oxidase (PPOX) catalyzes the aromatization of protoporphyrinogen IX into PpIX (**Figure 3**). PpIX can either be transformed into heme via ferrochelatase (FECH) or exported primarily through the ABCG2 transporter, which reduces intracellular PpIX levels by mediating its efflux (**Figure 3**). Mitoferrin (MRFN) mediates the transport of ferrous iron (Fe²^+^) into the mitochondrial matrix, providing the essential substrate for the terminal step of heme biosynthesis. Frataxin (FRTX) subsequently delivers Fe²^+^ to FECH, which catalyzes its incorporation into PpIX. This reaction generates heme, a non-fluorescent molecule that lacks photosensitizing activity due to the paramagnetic nature of the iron atom. In neoplastic cells, enzymatic imbalances, such as reduced FECH activity or accelerated 5-ALA uptake, lead to the selective accumulation of PpIX, providing the biochemical basis for 5-ALA-mediated PDT and fluorescence-guided resection (8, 62, 63).

Heme oxygenase-1 (HO-1) plays a pivotal role in the heme metabolism by catalyzing the degradation of heme into biliverdin, free iron, and carbon monoxide (CO)—**Figure 3**. This catabolic pathway is essential for maintaining cellular homeostasis, as excess free heme is highly cytotoxic due to its capacity to generate ROS. Consequently, iron metabolism is essential for heme production and is coordinated by specific mitochondrial proteins. In contrast to FRTX, downregulation of mitoferrin 2 (MRFN2) plays a more significant role in limiting heme production in tumor cells, even in the presence of exogenously supplied iron (63).

Although 5-ALA is consistently hydrophilic, its limited ability to penetrate hydrophobic barriers restricts its effectiveness in topical applications. To enhance the efficacy and selectivity of PDT, several derivatives of 5-ALA have been developed: the esterified derivatives, methyl aminolevulinate (MAL) and hexyl aminolevulinate (HAL). They differ significantly in their photochemical and photophysical predicted and experimentally tested properties (see **Supplementary Table**). 5-ALA is hydrophilic with a low lipophilicity (log P: -1.52), while MAL is slightly more lipophilic (log P: -1.2), and HAL is highly lipophilic (log P: 1.84), enabling deeper tissue penetration. HAL can lead to the highest PpIX formation efficiency, requiring significantly lower concentrations to achieve maximal PpIX accumulation (C_opt_) compared to 5-ALA and MAL. Additionally, HAL undergoes faster enzymatic cleavage, which contributes to its superior PpIX formation rate (64).

All three compounds exhibit optimal PpIX formation at physiological pH levels (7.0–7.6) according to *in vitro* experiments. However, at concentrations exceeding C_opt_, all three compounds reduce cell viability, highlighting the importance of precise dosing for effective PDT. Overall, HAL shows the most promising characteristics for enhanced PpIX-mediated PDT. While lower concentrations (e.g., 0.1-0.25 mM) reached a plateau after prolonged exposure, higher concentrations (e.g., 1.5 mM) exhibited a sustained linear increase in PpIX formation with extended drug exposure (64).

MAL, a more amphiphilic derivative, exhibits enhanced tissue penetration and requires shorter incubation times, showing promising outcomes in superficial lesions and currently undergoing evaluation in cervical neoplasia. HAL, the hexyl ester of ALA, demonstrates even greater membrane permeability but reduced hydrophilicity, resulting in higher intracellular accumulation of PpIX accumulation. HAL is already approved for photodynamic diagnosis in bladder cancer and is being investigated for emerging applications in gynecological oncology (8).

Despite promising clinical evidence demonstrating superior efficacy over conventional therapies, certain PDT protocols are associated with relapse, underscoring the need to understand mechanisms of resistance in cervical neoplastic cells (49, 54, 65). The therapeutic efficiency of 5-ALA-PDT can be influenced by various factors, including the depth of action, local physiological conditions, and the protocol of 5-ALA application. Persistent hrHPV infection remains a significant challenge, as it is closely linked to recurrence, emphasizing the importance of follow-up and monitoring of viral load post-treatment (8, 66).

Additionally, molecular mechanisms, such as high methylation levels of PAX1 genes (*PAX1hm*), have been shown to correlate with lower therapeutic efficiency and higher recurrence rates, highlighting the need to address these cellular resistance factors (55). Suboptimal PDT protocols, including inadequate 5-ALA concentration, application time, or insufficient treatment sessions, may result in poor PpIX accumulation and ineffective therapy, further contributing to relapse (67).

Optimizing 5-ALA-PDT for cervical intraepithelial neoplastic lesions requires not only refinement of treatment protocols but also a deeper understanding of the biological determinants of therapeutic resistance. Dysregulation of heme biosynthetic enzymes, perturbations in iron transport and delivery, and variability in porphyrin transporter activity profoundly influence intracellular PpIX accumulation and subsequent phototoxicity. Elucidating these mechanisms is essential to enhance treatment efficacy, minimize recurrence, and ensure durable long-term outcomes in the management of cervical neoplastic disease.

In the following section we critically examine the functional mechanisms of 5-ALA-derived PpIX photosensitization and the effects on mitochondrial structure and function, evaluates experimental evidence linking mitochondrial photodamage to apoptotic and non-apoptotic death pathways, and discusses the therapeutic and reproductive implications of mitochondrial-targeted PDT in cervical intraepithelial neoplasia.

### 4.2 The mechanistic landscape of 5-ALA-derived PpIX photoinactivation

Based on the mechanistic framework proposed by Bastos et al. (42), the action of PpIX derived from 5-ALA or its lipophilic esters (e.g., MAL or HAL) (42). 5-ALA-derived PpIX efficiently sensitizes cells to photoinactivation, with the amount of porphyrin directly correlating with the degree of cell killing, and this photosensitization proceeds via multiple concurrent mechanisms, (both type I and type II). Depending on the local microenvironment and the presence of suitable co-substrates, PpIX can adopt more specific roles: when it undergoes a closed redox cycle with endogenous electron donors (e.g., NADPH or ascorbate) and is regenerated per turnover while producing ROS in stoichiometric fashion, it acts as a photocatalyst; when it is consumed to generate chain-propagating radicals-particularly via Type I reactions that initiate lipid peroxidation cascades in membranes, it behaves as a photoinitiator; and when it is both regenerated (e.g., by redox cycling with cellular reductants) and simultaneously initiates radical chain reactions, it qualifies as a photocatalytic initiator. Because of these specific modalities whether PpIX can operate as a photocatalyst, a photoinitiator, or a dual-action photocatalytic initiator, the precise intracellular location of PpIX becomes a critical determinant of therapeutic efficacy. Consequently, optimizing photodynamic outcomes requires more than just maximizing overall cellular accumulation; it demands the strategic positioning of the PS within key subcellular compartments. The following section explores how directing 5-ALA-derived PpIX to specific organelles exploits these distinct photochemical behaviors to maximize targeted cell death.

### 4.3 Targeting organelles to 5-ALA-derived PpIX mediated PDT

Effective 5-ALA-PDT largely depends on the accumulation of PpIX in target tissues, which relies on the intricate balance of its uptake, metabolism, and efflux. Although lipophilic PpIX can redistribute to the endoplasmic reticulum (ER) or positioned in the plasma membrane, mitochondria play a central role in mediating the cytotoxic effects of 5-ALA-PDT.

**Figure 4:**
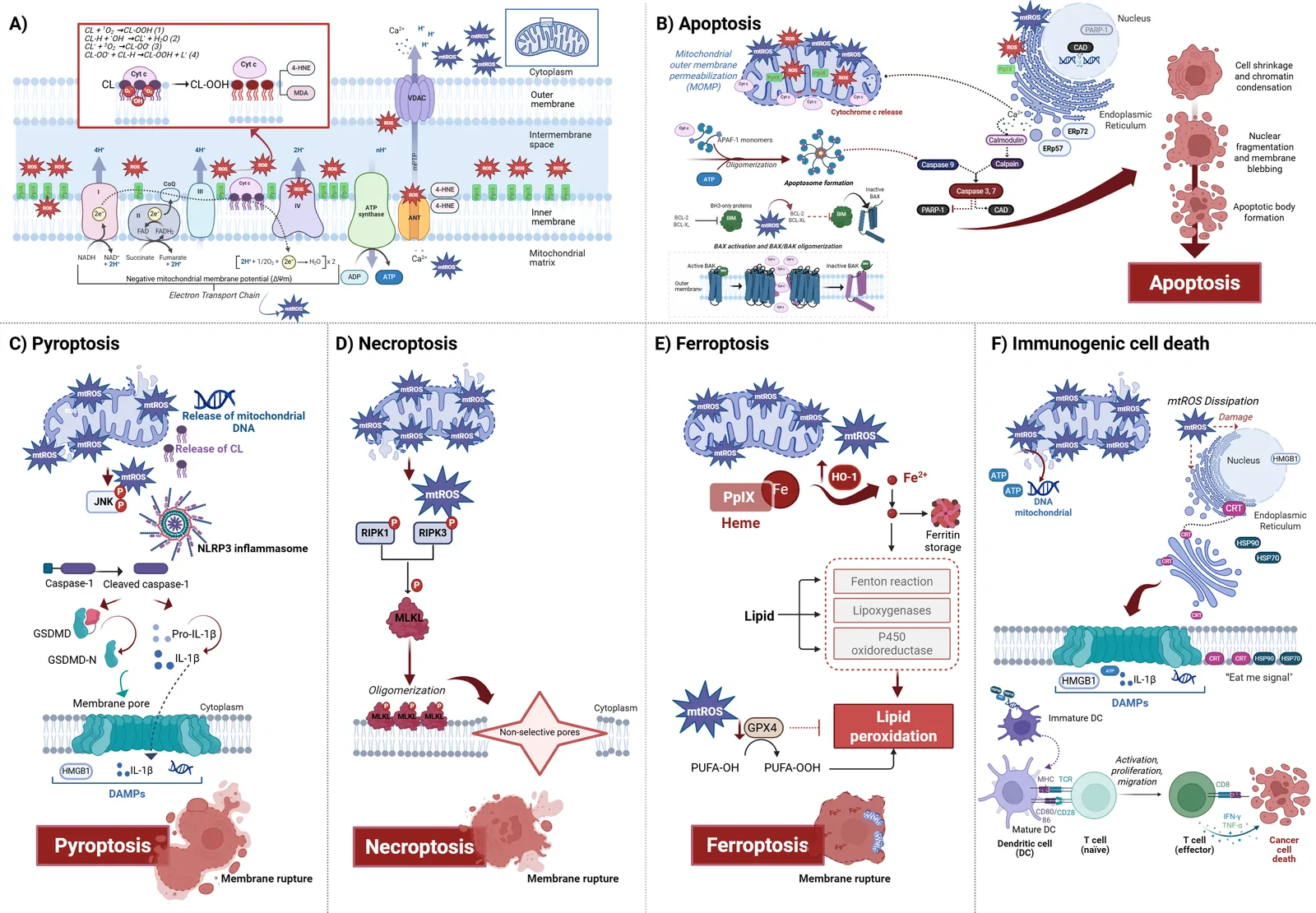
Mechanistic landscape of cell death modalities elicited by 5-aminolevulinic acid (5-ALA)-mediated photodynamic therapy. The schematic illustrates the integrated pathways of cell death and immune activation triggered by 5-ALA-PDT. **(A)** Mitochondrial oxidative stress and ETC dysfunction. PDT-induced generation of ROS, particularly ^1^O_2_ and hydroxyl radical (^•^OH), leads to the peroxidation of CL in the IMM. This oxidative damage disrupts the electron transport chain (ETC) at complexes I, III, and IV, leading to a loss of ΔΨm and the release of pro-apoptotic factors through VDAC and ANT. **(B)** Orchestration of apoptosis. Oxidative damage to CL into CL-OOH facilitates the detachment and cytosolic release of Cyt *c*. This is accompanied by a shift in the BCL-2 family balance (downregulation of BCL-2/BCL-X_L_ and upregulation of BIM), promoting BAX/BAK oligomerization and the subsequent activation of the Caspase-9/3/7 axis and PARP1 cleavage. Parallel pathways involve ER stress triggered by ROS and mtROS, leading to disruption of calcium-binding chaperones (e.g., ERp57 and ERp72), elevated cytosolic Ca^2+^ levels and activation of calpain, culminating in chromatin condensation, nuclear fragmentation, and the formation of apoptotic bodies. **(C)** Pyroptosis and inflammasome activation. Mitochondrial damage and mtROS release trigger the JNK signaling pathway and the assembly of the NLRP3 inflammasome. This activates caspase-1, which cleaves GSDMD. The N-terminal fragments of GSDMD form large trans-membrane pores, leading to the secretion of pro-inflammatory cytokines (IL-1β) and DAMPs, resulting in osmotic swelling and membrane rupture. **(D)** Necroptosis signaling. In specific cellular contexts, mtROS promote the phosphorylation and activation of the RIPK1/RIPK3 complex. This complex recruits and phosphorylates MLKL, which undergoes oligomerization and translocates to the plasma membrane, forming non-selective pores that facilitate necrotic cell rupture. **(E)** Ferroptosis and lipid peroxidation. 5-ALA-PDT enhances the accumulation of PpIX and subsequent heme degradation via HO-1, increasing the intracellular pool of labile iron (Fe^2+^). This iron catalyzes the Fenton reaction, driving the peroxidation of PUFAs. The overwhelming GPX4 antioxidant capacity and the accumulation of lipid hydroperoxides (PUFA-OOH) lead to catastrophic membrane failure. **(F)** Induction of ICD. The combined stress response induces the surface exposure of CRT and the extracellular release of HMGB1, ATP, and mtDNA. These DAMPs serve as “eat-me” and “find-me” signals for immature DCs. Upon maturation, these DCs (CD80+/CD86+) present tumor-associated antigens to naïve T cells, prompting the activation and infiltration of CD8+ effector T cells for antitumor immune response within the tumor microenvironment characterized by IFN-γ and TNF-α secretion. ANT, adenine nucleotide translocator; BAK, Bcl-2 homologous antagonist/killer; BAX, Bcl-2-associated X protein; BCL-2, B-cell lymphoma 2; BCL-X_L_, B-cell lymphoma-extra-large; BIM, Bcl-2-interacting mediator of cell death; CAD, caspase-activated DNase; CRT, calreticulin; Cyt c, cytochrome c; DAMPs, damage-associated molecular patterns; DCs, immature dendritic cells; ER, endoplasmic reticulum; ETC, electron transport chain; GPX4, glutathione peroxidase 4; GSDMD, gasdermin D; HMGB1, high mobility group box 1; HO-1, heme oxygenase-1; ICD, immunogenic cell death; JNK, c-Jun N-terminal kinase; MLKL, Mixed Lineage Kinase Domain-Like Pseudokinase; MOMP, mitochondrial outer membrane permeabilization; mtROS, mitochondrial reactive oxygen species; PARP-1, poly(ADP-ribose) polymerase 1; PUFAs, polyunsaturated fatty acids; RIPK, receptor-interacting protein kinase; ROS, reactive oxygen species; VDAC, voltage-dependent anion channel. Created in https://BioRender.com.

Mitochondria play a crucial role in cellular energy production through oxidative phosphorylation, nucleotide metabolism, amino acid and fatty acid metabolism, and even in regulating apoptosis or mitophagy. They consist of two membranes: the outer membrane (OMM) and the inner membrane (IMM), which delimit the intermembrane space and matrix (68). OMM and IMM differ regarding the protein/lipid ratio. IMM shows a higher protein level and a lower lipid content than OMM, mainly the mitochondria-specific phospholipid cardiolipin (CL) and phosphatidylethanolamine (PE), which are crucial for the activity of several mitochondrial proteins, including adenine nucleotide translocator and adenosine triphosphate (ATP) synthase (69)—**Figure 4A**.

The IMM contains the electron transport chain that generates ATP by oxidative phosphorylation (OXPHOS) and establishes a negative mitochondrial membrane potential (ΔΨm), typically ranges between -150 mV to -180 mV under normal physiological conditions (**Figure 4A**). Because of their central role in energy production and apoptosis regulation, mitochondria have long been considered primary targets for PDT (68).

ATP production can be disrupted by oxygen radicals originating from both internal and external sources. While mitochondria play a crucial role in maintaining cellular homeostasis, they are also a significant source of ROS. These mitochondrial ROS (mtROS) can damage mitochondrial components, such as lipids, proteins, and DNA, potentially leading to mitochondrial dysfunction and cell death, especially when antioxidant defenses are depleted or under conditions of oxidative stress. Key mtROS include O_2_^•-^, produced at complexes I and III of the electron transport chain, and H_2_O_2_, formed by the action of superoxide dismutase (SOD) on O_2_^•-^. Additionally, ^•^OH arise from H_2_O_2_ via the Fenton reaction, while peroxynitrite (ONOO^−^) is produced from the interaction of superoxide with nitric oxide (NO) (70).

PpIX-metabolized from 5-ALA primarily localizes in the mitochondria due to its lipophilic nature, interacting with the IMM (71). This effect is particularly relevant in the context of cervical lesions and cervical cancer related to hrHPV. hrHPV proteins can alter the function of different intracellular molecules and organelles, such as mitochondria, promoting cell proliferation, evasion of cell death, and genomic instability (72). Thus, the selective accumulation of PpIX within the mitochondria of HSIL creates a site-specific metabolic vulnerability.

The manner of cell death can vary depending on several factors, including cell type, treatment conditions, light dosage, and duration of exposure. For example, depending on 5-ALA concentration used, tumor cells may undergo alternative forms of cell death, such as apoptosis or necrosis. At lower concentrations, cells undergo inflammatory cell death, whereas higher concentrations induce apoptosis or necrosis (73). **Figure 4** illustrates the primary mechanisms of cell death induced by 5-ALA-PDT.

The efficacy of 5-ALA-PDT is driven by the generation of ROS through Type I (radicals) and Type II (^1^O_2_) photochemical pathways. While ROS production typically scales with 5-ALA concentration until enzymatic saturation, therapeutic outcomes are strictly constrained by three factors: (1) incubation kinetics, balancing cellular uptake against metabolic PpIX efflux (74); (2) oxygen availability, where tumor hypoxia limits ROS yield (75); and (3) photobleaching or self-quenching, which can paradoxically diminish energy transfer at excessive PpIX concentrations (74).

Primary PpIX-photoinduced ROS leads to immediate oxidative damage to critical mitochondrial components, including CL in the IMM and the proteins of the OXPHOS complexes (**Figure 4A**). This disruption of OXPHOS boosts the production of mtROS, leading to further oxidative damage to mitochondrial lipids, proteins, and DNA, ultimately impairing mitochondrial function and triggering cell death. The process begins when PpIX-photoinduced species attack the polyunsaturated fatty acid residues of CL, which is uniquely characterized by its dimeric structure containing four polyunsaturated fatty acid side chains—most commonly linoleic acid (C18:2). ¹O_2_ interacts directly with double bonds to form primary allylic hydroperoxides—CL-OOH (1), while Radical species, such as O_2_^•-^ and ^•^OH, abstract a hydrogen atom from the α-methylene carbon of the linoleic acid residue of CL (CL-H) to form a carbon-centered lipid radical—CL^•^ (2). The lipid radical rapidly reacts with ^3^O_2_ to form a peroxyl radical—CL-OO^•^ (3). This peroxyl radical then abstracts a hydrogen from a neighboring CL-H, creating CL-OOH (4) and a new lipid radical to propagate the cycle (**Figure 4A**). This process facilitates a self-sustaining propagation phase where the resulting radical interacts again with ^3^O_2_ to form peroxyl radicals (3), which systematically oxidize adjacent side chains into lipid hydroperoxides CL-OOH (4). The accumulation of lipid hydroperoxides CL-OOH disrupts the electrostatic and hydrophobic interactions between CL and cytochrome c (Cyt *c*), facilitating its release into the cytosol (69)—**Figure 4A**.

Persistent oxidative stress and the presence of transition metals (like iron) lead to β-cleavage of CL-OOH into reactive aldehydes, notably 4-hydroxy-2-nonenal (4-HNE) and malondialdehyde (MDA). 4-HNE and MDA are “second messengers” of oxidative stress because they have a longer half-life than free radicals and can diffuse to hit targets further away. Reactive aldehydes like 4-HNE can modify mitochondrial protein structures by forming covalent adducts with nucleophilic residues (specifically lysine, histidine, and cysteine residues) via Michael addition or Schiff base formation (76). These structural changes may alter protein-protein interactions, including those involving components of the Mitochondrial Permeability Transition Pore (mPTP), promoting pore opening (69, 70) (**Figure 4A**).

mPTP is a multiprotein complex located at the junction of the inner and outer mitochondrial membranes (**Figure 4A**). Its composition is not fully defined, but key components include adenine nucleotide translocator (ANT), ATP synthase, phosphate carrier (PiC), and cyclophilin D (Cyp-D), which regulates its opening. As a non-selective channel, it plays a critical metabolic switch: transient, reversible opening supports calcium (Ca^2+^) homeostasis and oxidative stress mitigation, whereas sustained opening inevitably triggers cell death. Its activation is tightly regulated by Ca^2+^ levels, mtROS, and shifts in ΔΨm (70).

The initial oxidative insult triggered by 5-ALA-PDT acts as a trigger for a catastrophic physiological mechanism known as ROS-induced ROS release, resulting in a ROS burst that increases the mPTP opening, allowing for the rapid efflux of accumulated endogenous mtROS into the vicinity of neighboring mitochondria (**Figure 4A**). It creates a positive-feedback loop that propagates an “avalanche-like” release of mtROS throughout the cell. Beyond this mtROS dissipation, PPIX can migrate to the ER, where its photoactivation disrupts calcium-binding chaperones (e.g., ERp57 and ERp72), causing Ca^2+^ release into the cytosol. Elevated cytosolic Ca^2+^ levels activate calpain, which further stimulate caspase-3 and calcium-dependent endonucleases, amplifying apoptotic signals. Additionally, ER stress leads to the accumulation of misfolded proteins, triggering the unfolded protein response and further promoting apoptosis (77). Thus, combining mitochondrial dysfunction and ER stress we can overcome cellular survival mechanisms in tumor cells (**Figure 4B**).

Upon photoinduced oxidative stress cells downregulate BCL-2 and BCL-X_L_ expression, thereby liberating BIM to bind to the surface of inactive, monomeric BAX (in the cytosol) or BAK (already on the mitochondrial membrane) – **Figure 4B**. Once “activated” by a BH3 protein (BIM), BAX and BAK oligomerize and mediated Mitochondrial Outer Membrane Permeabilization (MOMP) pores (**Figure 4B**). This process allows the leakage of intermembrane space components and Cyt *c*. Once released into the cytosol, Cyt *c* binds to APAF-1, triggering a conformational change and the assembly of the heptameric apoptosome (**Figure 4B**). This complex recruit and activates pro-caspase-9, which subsequently initiates an executioner cascade by proteolytically cleaving caspase-3 and -7. These events result in DNA fragmentation by caspase-activated DNase (CAD), cleavage of Poly [ADP-ribose] polymerase 1 (PARP-1), and cellular disassembly. By cleaving PARP1, the cell prevents ATP depletion, thereby preserving sufficient energy to execute an orderly, non-inflammatory apoptotic program (77–81). This apoptotic response can be further enhanced by FECH inhibitors (82). Without regulation, BAX/BAK proteins further assemble into large-scale “megapores”. These expansive structures permit the escape of mitochondrial DNA (mtDNA), which may subsequently trigger inflammatory signaling pathways upon entering the cytoplasm (83).

5-ALA-PDT may lead to mtROS that disrupt mitochondrial membrane integrity, causing the externalization and release of CL and mitochondrial DNA (mtDNA) (**Figure 4C**). Together, mtROS, mtDNA and released CL act as potent damage-associated molecular patterns (DAMPs) that may directly activate the NLRP3 inflammasome (73). This mtROS accumulation can also play a critical role in activating the JNK signaling pathway, which subsequently promoted NLRP3 inflammasome assembly. This protein complex recruits and activates caspase-1, which proteolytically processes pro-inflammatory cytokines (IL-1β) and cleaves gasdermin D (GSDMD). The N-terminal fragments of GSDMD then form large pores in the plasma membrane, culminating in pyroptosis—a highly inflammatory form of programmed cell death that alerts the systemic immune response to the tumor site (84, 85). The 5-ALA-PDT-induced pyroptosis can also release other inflammatory mediators like the non-histone nuclear DNA-binding protein HMGB1 that regulates transcription and organization of DNA.

While pyroptosis (driven by caspase-1) and apoptosis (driven by caspases-3, 7, 9) are the most common outcomes of 5-ALA-PDT, mtROS can indeed shift the cell toward necroptosis (**Figure 4D**). Elevated mtROS levels promote the oligomerization and autophosphorylation of RIPK1 and RIPK3 that phosphorylate MLKL. Phosphorylated MLKL moves from the cytosol to the plasma membrane, where it oligomerizes to form large, non-selective pores. Unlike the non-inflammatory apoptotic blebbing, these pores cause the cell to swell and explode, releasing all its contents—including the mtDNA, mtROS, and HMGB1 into the surrounding tissue (73). Inducing necroptosis via mtROS is often a goal in treating “apoptosis-resistant” tumors. If a cancer cell has mutated its caspases or overexpressed BCL-2, downregulated APAF-1 to the point that it can’t undergo normal apoptosis, the high-intensity mtROS can bypass those blocks and force the cell into necroptosis instead.

The PpIX-triggered mitochondrial dysfunction also induces ferroptosis, a unique form of programmed cell death dependent on iron, characterized by the accumulation of lipid peroxidation, which involves suppression of the enzyme GPX4 (glutathione peroxidase 4) plays a critical role in protecting cells from ferroptosis and HO-1 upregulation (86) (**Figure 4E**). This process has gained attention as a promising approach to treating tumor cells resistant to conventional therapies, as these cells are particularly vulnerable to oxidative stress caused by ferroptosis. In cervical cancer cells treated with ALA-based PDT, increased ROS and lipid ROS levels resulted in decreased expression of GPX4, a key protein that protects against ferroptosis (87, 88) (**Figure 4E**).

Mitochondrial PpIX photosensitization generates mtROS that propagates to the ER, triggering an Unfolded Protein Response (UPR) and culminating in immunogenic cell death (ICD) (**Figure 4F**). This “pre-apoptotic ER stress” drives the translocation of the chaperone calreticulin (CRT) from the ER to the plasma membrane. Surface-exposed CRT, along with heat shock proteins (HSP70/90), serves as a potent “eat-me” signal for antigen-presenting cells (APCs). The synergy between these ER-derived signals and mitochondrial/nuclear DAMPs (e.g., ATP and HMGB1) ensures an inflammatory rather than “silent” death. These molecular adjuvants stimulate the recruitment and maturation of dendritic cells (DCs)—evidenced by CD80+ and CD86+ upregulation, which ultimately prime a robust CD8^+^ T-cell response and the secretion of pro-inflammatory cytokines, specifically TNF-α and IFN-γ) (89). Thus, 5-ALA-PDT emerged as a promising therapeutic strategy to activate pyroptosis, ferroptosis, and immunogenic cell death, which are critical mechanisms for eliminating cancer cells and modulating tumor microenvironment (90).

Boosting the effectiveness of anticancer immunotherapy has been identified as a promising approach to deal with resistance to targeted therapies and chemotherapy in cervical cancer. Current biomarkers may predict immunotherapy sensitivity and prognosis (90, 91). Among these identified biomarkers, TFRC (Transferrin Receptor) and DMT1 (Divalent Metal Transporter 1) stand out as the most relevant for predicting the effectiveness of 5-ALA-PDT. TRFC and DMT1 play a critical role in cellular iron uptake, which is essential for heme biosynthesis, and its elevated expression has been associated with tumor progression and poor prognosis in cervical cancer (90, 91). Thereby, their expression levels could predict the efficiency of PpIX accumulation and, consequently, the effectiveness of 5-ALA-PDT. Additionally, checkpoint inhibitors, such as *TIGIT*, *CTLA4*, *BTLA*, *CD27*, and *CD28*, which are associated with enhanced immune response and better prognosis, may also be relevant for predicting the immunotherapeutic benefits of 5-ALA-PpIX-PDT (91). However, further research is needed to validate these biomarkers and elucidate their roles in the context of 5-ALA PpIX/PDT and ferroptosis.

While mitochondrial dysfunction induced by 5-ALA-PDT initiates a spectrum of regulated cell death pathways (e.g., apoptosis, ferroptosis, pyroptosis, and necroptosis), emerging evidence highlights autophagy as a critical cellular response to photodynamic stress. Understanding the interplay between autophagy and these regulated cell death pathways is crucial for optimizing PDT strategies and improving therapeutic outcomes.

The next sections explore how 5-ALA-PDT influences autophagic flux, the dual role of autophagy in cell survival and death, and its implications for therapeutic efficacy and resistance in hrHPV-associated cervical lesions.

### 4.4 Autophagy and 5-ALA-PDT

**Figure 5:**
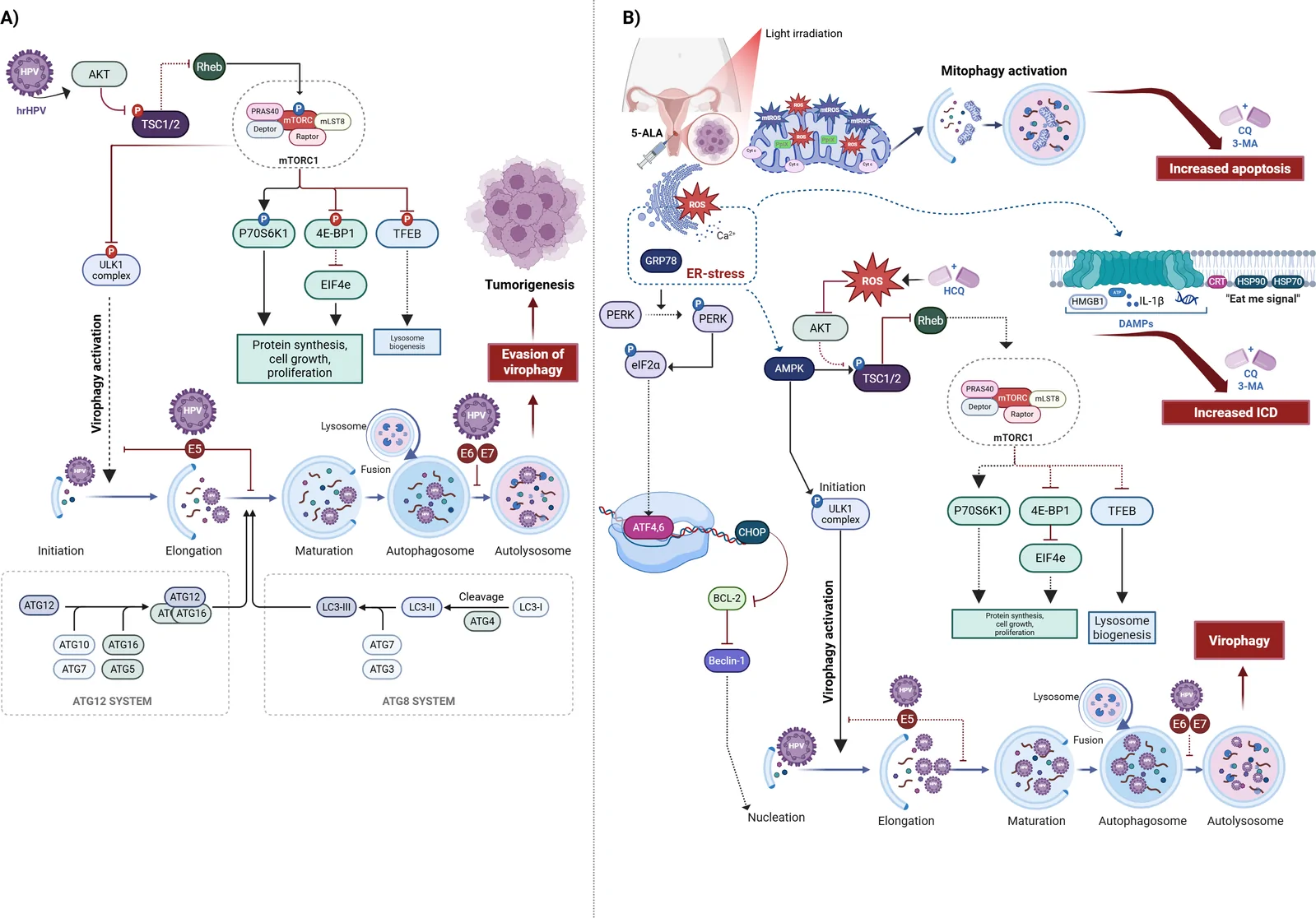
Mechanistic interplay between hrHPV pathogenesis and 5-ALA-PDT -induced stress. **(A)** hrHPV-mediated evasion of virophagy and oncogenic signaling: High-risk human papillomavirus (hrHPV) entry leads to AKT activation. AKT-mediated inhibition of the TSC1/2 GTPase-activating protein complex maintains the small GTPase Rheb in its active, GTP-bound state. Active Rheb subsequently triggers the kinase activity of mTORC1 at the lysosomal membrane. Upon mTORC1-mediated phosphorylation, the transcription factor EB (TFEB) is sequestered in the cytoplasm by 14-3-3 proteins, thereby repressing the transcription of genes involved lysosomal biogenesis and autophagy. Simultaneously, primary effectors of protein synthesis, such as S6K1 and 4E-BP1, are recruited to the active mTORC1 complex at the lysosomal surface. Active mTORC1 directly phosphorylates and inactivates the ULK1 complex at the lysosomal surface, inhibiting the autophagic flux. Viral oncoproteins also compromise autophagy—E5 inhibits phagophore elongation, while E6 and E7 prevent autophagosome-lysosome fusion. This multi-stage blockade facilitates the evasion of virophagy, promoting viral persistence and tumorigenesis. **(B)** Autophagy modulation and hrHPV clearance: 5-ALA-PDT generates acute reactive oxygen species (ROS), triggering a robust ER stress response. The PERK-CHOP Axis: Dissociation of GRP78 activates the PERK/eIF2α/ATF4/6 pathway, leading to the upregulation of CHOP. CHOP suppresses BCL-2 expression, relieving the inhibition of Beclin-1 to initiate nucleation and drive the transition toward apoptosis. ROS-induced AMPK activation antagonizes the viral-induced mTORC1 pathway, restoring autophagic initiation and promoting mitophagy. Inhibition of 5-ALA-PDT -induced mitophagy by chloroquine (CQ) prevents the clearance of damaged mitochondria, thereby sensitizing cells to apoptosis. Autophagy inhibitors – CQ or 3-methyl adenine (3-MA), synergistically increases the emission of damage-associated molecular patterns (DAMPs) and executes immunogenic cell death (ICD), effectively bypassing the hrHPV-mediated autophagic block to resolve viral load and suppress tumor growth. Created in https://BioRender.com.

Autophagy exerts a context-dependent dual role in cancer, either supporting or inhibiting tumor progression, and is classified into macroautophagy, microautophagy, and chaperone-mediated autophagy (CMA) (92). Particularly, macroautophagy is a cellular process in which cytoplasmic components, including damaged proteins and organelles, are sequestered within double-membraned autophagosomes. These structures originate from phagophores that elongate and close before fusing with lysosomes, where lysosomal hydrolases degrade the enclosed material (93). During viral infections a form of selective autophagy (virophagy) can be activated to degrade virus particles, promoting ICD, pro-inflammatory responses, and presenting antigens to T cells, aiding adaptive immunity. Thus, virophagy may play a crucial role in the host’s defense against hrHPV infections (94)—**Figure 5A**.

hrHPV has developed strategies to evade or inhibit virophagy to ensure its persistence and promote tumorigenesis. During initial infection, HPV16 binding and entry activate the EGFR/PI3K/AKT/mTOR pathway to suppress autophagy induction (95)—**Figure 5A**. Specific inhibitors of PI3K (e.g., LY294002) and mTOR (e.g., PP242) significantly reduce hrHPV infection (e.g., HPV16), demonstrating the critical role of this pathway in viral entry and replication (95).

The activation of mTOR leads to phosphorylation of mTOR complex 1 (mTORC1) substrates, including 4E-BP1 and S6K (95). Once activated via phosphorylation, these effectors dissociate and localize to ribosomes in the cytosol and endoplasmic reticulum (ER) to initiate the translation of biomass-building proteins. In the context of hrHPV infection, this anabolic shift not only drives cellular proliferation but also sustains the high translational demand required for viral protein synthesis and oncogenic progression.

Hyperactivation of mTORC1 at the lysosomal membrane can also exert a dominant inhibitory effect on transcription factor EB (TFEB), the master regulator of lysosomal biogenesis and autophagy (96). Upon mTORC1-mediated phosphorylation, TFEB can be sequestered in the cytoplasm by 14-3-3 proteins, thereby repressing the transcription of genes within the coordinated lysosomal expression and regulation (CLEAR) network—including *ATG7*, *ATG4*, Beclin-1 (*BECN1*), LC3 (*MAP1LC3A*), and *ULK1* (97–99). BECN1 and ATG7 are key components of the autophagy machinery, and downregulating these proteins resulted in increased HPV16 infection (95). Complementing its regulation of TFEB, active mTORC1 directly phosphorylates and inactivates the ULK1 complex at the lysosomal surface, thereby imposing a molecular blockade on the initiation of autophagosome formation and further consolidating the hrHPV-driven suppression of autophagic flux (**Figure 5A**). This process prevents the degradation of incoming viral particles in autophagosomes (95).

Consequently, the sustained activation of the PI3K/AKT/mTORC1 axis coupled with the systematic suppression of virophagy culminates in elevated viral load and the intracellular stabilization of the hrHPV oncoproteins E5, E6, and E7. Beyond their primary role in driving oncogenic transformation, these oncoproteins orchestrate a multi-stage blockade of autophagic flux, impairing both autophagosome biogenesis and lysosomal maturation (100, 101)—**Figure 5A**. Notably, the HPV16 E5 oncoprotein further compromises cellular homeostasis by inducing the transcriptional downregulation of a comprehensive suite of core autophagy-related (ATG) genes, including *BECN1, ATG5, MAP1LC3B (LC3), ULK1, ATG4A*, and *ATG7* (101). This systematic depletion of autophagic machinery effectively “blindfolds” the cell’s innate defenses, facilitating viral persistence and disease progression. Importantly, the autophagy-hrHPV interaction is context-dependent, exerting either protective or deleterious effects depending on the infection stage and lesion type (HSIL or CIS) (102).

Autophagy impairment was linked to the early development of LSIL by HPV16, evidenced by increased LC3 and accumulation of p62, indicating a blockage in the fusion of the autophagosome with the lysosome (103, 104). Thereby, the accumulated autophagic markers p62, LC3, and Beclin 1 in hrHPV-positive lesions could potentially serve as biomarkers of cervical disease. As the disease progressed to HSIL, MIC, and ICC, autophagy flux is normalized, with the formation of autophagolysosomes and reduced p62 levels. Autophagy inhibition with CQ reinforced the role of autophagy dysfunction in hrHPV-induced carcinogenesis (103). Indeed, as a self-degradative process, autophagy plays a dual role in cancer by either promoting cell survival under stress conditions or contributing to cell death depending on the tumor microenvironment (105). This activation is commonly associated with inhibition of AKT/mTOR pathway or activation of AMP-activated protein kinase (AMPK), which can be pharmacologically or photochemically modulated (105, 106). Following PDT-induced cellular stress, autophagy represents a context-dependent process that may exert either cytoprotective or cytotoxic effects, contingent upon the specific PS employed and the cellular phenotype (106). Rather than directly initiating cell death, autophagy frequently accompanies PDT-induced demise as an adaptive response, reflecting the cell’s attempt to mitigate oxidative stress and preserve survival capacity. Its inhibition with 3-Methyladenine (3-MA) disrupts this protective balance following 5-ALA-PDT by downregulating anti-apoptotic proteins such as BCL-2 and survivin, while simultaneously upregulating pro-apoptotic mediators including BAX. This molecular shift enhances tumor susceptibility to photo-induced stress, resulting in apoptosis and necrosis (81). Besides, by promoting mitochondrial dysfunction and ATP depletion, 5-ALA-PDT activates AMPK and leads to autophagy-mediated death. Autophagy inhibitors like 3-MA can partially prevent this cell death, highlighting AMPK’s role in PDT-induced autophagy and its potential for cancer therapy (107).

Following PDT, cells trigger organelle-specific macroautophagy—notably mitophagy and reticulophagy, to sequester and degrade oxidized mitochondria and ER (106, 108). This potent induction of autophagic flux by 5-ALA-PDT may bypass the hrHPV-mediated inhibitory signaling, restoring cellular homeostasis and suppressing oncogenic progression (**Figure 5B**).

5-ALA-PDT activates autophagy through the RAS/RAF/MEK/ERK pathway while suppressing the PI3K/AKT/mTOR pathway. This dual modulation contributes to the therapeutic effects of ALA-PDT in reducing viral load in hrHPV-positive patients (109). The PDT-triggered AKT inhibition alleviates the mTOR-mediated suppression of autophagy and lysosome biogenesis, while simultaneously facilitating the induction of apoptosis (**Figure 5B**). Treatment with hydroxychloroquine (HCQ) enhances the efficacy of 5-ALA-PDT by increasing ROS levels and amplifying the antiproliferative effects on hrHPV-infected cells (109).

5-ALA-PDT induces ER-stress in hrHPV-infected cells, orchestrating a dual response of autophagy and apoptosis. Mechanistically, this stress promotes the efflux of Ca^2+^ from the ER lumen into the cytosol, activating the CaMKKβ/AMPK signaling axis, AMPK pathway, which inhibits mTOR activity to promote autophagy as a survival mechanism (110). Within the Unfolded Protein Response (UPR), GRP78 serves as an early-stage initiator by binding to transmembrane proteins such as PERK under normal conditions, keeping them inactive. During ER stress, the accumulation of misfolded or unfolded proteins in the ER lumen leads to the dissociation of GRP78 from PERK, allowing PERK activation. Activated PERK phosphorylates eIF2α, which reduces global protein synthesis and promotes the expression of stress-related genes such as ATF4,6. ATF4, along with ATF6, contributes to the upregulation of CHOP, a key pro-apoptotic transcription factor. As a terminal effector, CHOP executes the transition from an adaptive response to programmed cell death by repressing BCL-2, thereby facilitating MOMP. Furthermore, the downregulation of BCL-2 relieves its inhibitory sequestration of Beclin-1, effectively integrating autophagic signaling into the terminal ER-stress response and apoptosis (**Figure 5B**). Thus, 5-ALA-PDT could be particularly useful in addressing hrHPV’s effects on autophagy in the early infection or disease progression stages.

Autophagy serves as a cytoprotective mechanism during 5-ALA-PDT; its pharmacological inhibition via chloroquine (CQ) potentiates ICD and the subsequent immune cascade (**Figure 5B**). This synergistic approach enhances the emission of DAMPs (e.g., CRT, HSP70/90, ATP, and HMGB1), driving the maturation of DCs and, ultimately, a robust systemic anti-tumor response characterized by CD8+ cytotoxic T-cell infiltration and elevated secretion of pro-inflammatory cytokines, specifically TNF-α and IFN-γ (89).

Beyond pharmacological modulation of autophagy, prognostic models incorporating the downregulation of autophagy-related genes (e.g., *CHMP4C*, *FOXO1*, *RRAGB*) may inform personalized treatment strategies and enhance the effectiveness of 5-ALA-PDT in cervical cancer (111). This is particularly relevant given the interplay between hrHPV oncogenic activity and autophagy, as viral proteins can disrupt autophagic flux and thereby influence PpIX accumulation, phototoxicity, and cellular survival. By addressing hrHPV-induced autophagy dysregulation, such models hold promise for reducing recurrence rates and improving long-term outcomes following PDT.

5-ALA-PDT has been shown to alter cervicovaginal cytokine expression (e.g., IL-6 and IL-8) in patients with hrHPV and LSIL (112). This immunomodulatory effect could potentially enhance the body’s natural defense mechanisms against hrHPV infection and its impact on cellular processes like autophagy.

Overall, PDT’s ability to restore autophagy and induce apoptosis in hrHPV-infected cells by modulating key pathways, such as PI3K/AKT/mTOR and AMPK, not only reduces viral load but also disrupts hrHPV’s manipulation of host cell processes. Its selectivity for transformed cells and immunomodulatory effects further highlight its therapeutic promise, especially in early-stage disease. However, despite these advances, important questions remain regarding the precise mechanisms by which hrHPV modulates different forms of autophagy, such as mitophagy, and how these interactions influence treatment outcomes (113, 114). These gaps underscore the need for further research to optimize PDT strategies and fully understand their impact on hrHPV-associated disease.

## 5 Mechanisms of resistance to 5-ALA-PDT

A primary therapeutic challenge in high-grade lesions involves the eradication of cancer stem-like cells (CSCs) and latent hrHPV reservoirs located within the cervical epithelial basal layer. The cancer stemness-5-ALA–PDT resistance is driven by ABCG2-mediated PpIX efflux, enhanced stress responses, autophagy, and reduced ROS levels (115, 116). In addition, elevated ABCG2 activity, frataxin, and FECH expression increase mitochondrial iron and accelerate PpIX conversion to heme, diminishing 5-ALA-PDT sensitivity (117). Although these cervical CSCs may elicit the efflux of PpIX and mediating relative PDT resistance, optimized light fluences and extended PS incubation profiles can overwhelm these efflux dynamics. Direct photodynamic action triggers a profound surge in localized exogenous ROS that damages the protective pericellular niche, disrupting the stromal-epithelial crosstalk essential for stemness maintenance. Crucially, because 5-ALA and its ester derivatives selectively accumulate within the hypermetabolic, hrHPV-transformed dysplastic epithelium down to the basement membrane, PDT achieves targeted ablation of infected basal cells. Unlike conventional excisional procedures that strip the underlying stroma, PDT preserves the extracellular matrix architecture, allowing uninfected, quiescent resident stem cells to subsequently proliferate and safely regenerate a functional, intact cervical epithelium. Specific ABCG2 inhibitors, such as fumitremorgin C, Ko143, and lapatinib, have been shown to increase ALA-induced PpIX accumulation and photodamage, with lapatinib and Ko143 producing dose-dependent improvements in fluorescence and PDT response (118).

Non-stem cells, such as dormant cancer cells (DCCs), exhibit upregulated PEPT1 and ABCB6 with downregulated ABCG2, favoring PpIX retention, alongside metabolic shifts such as increased acyl-CoA synthetases that support porphyrin metabolism (61, 119). These features highlight metabolic determinants as potential biomarkers of 5-ALA-PDT responsiveness.

Cancer metabolism further shapes PDT efficacy: the Warburg effect impairs heme biosynthesis by limiting NADH availability and reducing FECH activity (120). Dysregulation of enzymes, transporters (e.g., ABCB6, ABCG2), and iron-handling proteins, including mitoferrins (e.g. MRFN1, MRFN2), FECH, HO-1/HO-2, and CPOX, critically modulates PpIX synthesis, conversion, and efflux (63, 118, 121–124). Increased expression of either ABCB6 or biosynthetic enzymes (e.g., CPOX) enhances also ALA-induced PpIX accumulation and photodamage (123, 124). Notably, FECH inhibition by iron chelators such as desferrioxamine (DFO) markedly increases PpIX accumulation and light-induced cytotoxicity (122).

Collectively, these findings underscore mitochondrial iron metabolism and heme biosynthesis as central targets to enhance 5-ALA–PDT. Their modulation not only supports improved therapeutic selectivity but also strengthens the clinical application of δ-aminolevulinates in PDT and fluorescence-guided procedures (125). This metabolic framework provides the rationale for subsequent exploration of mitochondrial targeting strategies in cervical intraepithelial neoplasia.

## 6 PDT as strategy to treat cervical intraepithelial neoplasias

Cumulative clinical evidence underscores the efficacy of 5-ALA-mediated PDT in the management of cervical intraepithelial neoplasia. In clinical trial settings, complete response (CR) is typically characterized by a composite endpoint comprising three elements: histological regression (the absence of dysplastic or atypical epithelial proliferation), cytological normalization—negative ThinPrep cytology test (TCT), and the absence of suspicious lesions during colposcopic evaluation (126, 127). Furthermore, virological clearance—defined as the conversion from a positive baseline hrHPV status to a negative result during post-treatment follow-up, is a critical metric for long-term success (128).

### 6.1 Therapeutic outcomes in low-grade and high-grade lesions

CIN1 often result from transient hrHPV infections. While most cases are resolved naturally, approximately 30% persist, 10% progress to HSIL within two years, and 5% advance to ICC. A retrospective study with 751 women with a cytological diagnosis of LSIL (CIN1) concluded that the risk of developing CIN3 is not determined simply by having CIN1. Instead, the real risk factor is whether the woman has a persistent infection with certain high-risk HPV types — specifically HPV 16, 18, 31, or 33. On this direction, to treat persistent CIN1 is crucial for cervical cancer prevention (129).

Trials using 5-ALA (10–20% w/w) with 635 nm irradiation at 100 J/cm² have reported CR rates of 84.5–93.1% and hrHPV clearance rates of 76.9–81.8% for CIN1 at 6–12 months (51–53, 130). Compared to routine follow-up, 5-ALA-PDT significantly reduces the rate of persistent lesions (19.6% vs. 39.0%) and effectively prevents progression to HSIL. Notably, younger cohorts (<45 years) demonstrate significantly higher hrHPV clearance rates at 12 months compared to older patients (83.4% vs. 61.1%), suggesting that age-related immune proficiency may augment therapeutic outcomes (53).

Comparable efficacy was observed in CIN2–3, with CR rates ranging from 77.8% to 97% and hrHPV clearance between 64.4% and 92.5% (54–58, 130–135). While persistent or residual lesions, occasional recurrence, and progression to higher HSIL grades occur in 7.1–42%, 2.3-3.3%, and 25% of cases, 5-ALA-PDT has emerged as a safe and effective alternative for treating positive HSIL margins (49, 54, 58, 131). Unlike repeated conization, PDT offers a superior fertility-sparing strategy by preserving cervical anatomical integrity and significantly reducing recurrence rates (3.3% vs. 6.7% in surgical cohorts) at 2-year follow-up (58).

Recent comparative studies indicate that 5-ALA-PDT is non-inferior to LEEP for CIN2 management. Both modalities demonstrate similar pathological regression (90–97%) and hrHPV clearance (79–90%) at 6 to 18 months (133, 134). However, some prospective data suggest 5-ALA-PDT may yield superior remission (83.3% vs. 64.7%) and virological clearance (78.6% vs. 60.3%), particularly for high-risk genotypes HPV16, 18 (135). A meta-analysis of 851 patients further reinforces that hrHPV clearance in PDT cohorts may surpass that of conventional surgical excision (136).

The regression of CIN2 lesions and subsequent hrHPV clearance are primarily driven by the activation of ICD and localized immune responses. Mechanistically, 5-ALA-PDT enhances the cytotoxic activity of CD8+ T cells by upregulating the expression of granzyme B and chemokine receptor 3 (CXCR3) while simultaneously downregulating programmed cell death protein 1 (PD-1) to prevent immune exhaustion (7). These pathways collectively facilitate the targeted elimination of hrHPV-infected cells and the regression of precancerous lesions.

Despite its high efficacy, the therapeutic reach of PDT is constrained in cases involving the endocervical canal or deep glandular involvement. For instance, CIN3 with glandular involvement shows significantly lower hrHPV clearance rates (63.16%) compared to lesions without such involvement (92.6%) (54). Furthermore, HSIL persistence is observed in 42.9% of patients with cervical canal involvement (55). Optimizing outcomes in these scenarios requires meticulous patient selection, prolonged irradiation protocols for deeper tissue penetration, and rigorous follow-up (132).

Alternative photosensitizer esters, such as MAL and HAL, have also shown promise for CIN2 treatment (137, 138). While HAL-based formulations (e.g., APL-1702) demonstrate significant histological regression in CIN2/3 and HPV16, 18 clearance, their CR rates in some reports remain lower than those of 5-ALA gel formulations (138, 139). Recent studies also emphasize that the interval between sessions is critical; for example, two sessions at 21-day intervals may enhance cytological remission in high-grade CIN (66).

Collectively, these trials consistently demonstrate that 5-ALA-PDT achieves promising rates of lesion remission and hrHPV clearance across CIN1–3, with efficacy comparable or superior to excisional methods while preserving cervical integrity and reproductive outcomes. Recent advances have focused on optimizing PS formulations, delivery systems, and combination strategies to further enhance selectivity, immune activation, and long-term disease control (7, 8).

To confirm the clinical durability of 5-ALA-PDT beyond standard short-term intervals, longitudinal cohort studies have tracked patients for periods extending from 2 to 5 years (130). Accumulating data demonstrates that achieving CR and virological clearance at 2-year post-treatment window correlates with long-term lesion-free survival, with recurrence rates stabilizing less than 3%, a profile highly competitive with LEEP (58).

Mechanistically, this exceptional long-term efficacy is driven by a dual-action therapeutic cascade dominated by apoptosis and ICD (see **Figure 4**). While emerging cell death pathways such as ferroptosis, pyroptosis, and necroptosis have been identified in preclinical models (87–90), apoptosis and ICD possess the most robust, mature experimental evidence in cervical PDT (7, 73) Because PpIX preferentially partitions into the mitochondria and endoplasmic ER, light activation prompts the canonical intrinsic caspase-9/caspase-3 apoptotic cascade. Concurrently, acute ER stress triggers the rapid cell-surface translocation of CRT alongside the extracellular release of ATP, HMGB1, and heat shock proteins (HSP70 and HSP90) – see **Figure 4F**. These damage-DAMPs transform the immunosuppressive, hrHPV-altered microenvironment into an immunogenic milieu, actively recruiting and priming tumor-specific cytotoxic CD8+ T cells to eliminate adjacent latent viral reservoirs and prevent late disease recurrence.

### 6.2 Clinical implementation, patient selection, and guideline recommendations

Successful translation of cervical PDT from clinical trials to standard gynecologic oncology pathways necessitates rigid patient selection criteria and standardized delivery frameworks. The ideal target demographic comprises reproductive-aged women (<40 years) diagnosed with histologically confirmed CIN2 or localized, non-invasive HSIL, who express a strong desire for fertility preservation and wish to avoid the anatomical and obstetric sequelae of excisional surgery (e.g., cervical stenosis, cervical incompetence, and subsequent preterm delivery).

Clinical eligibility requires a fully visible transformation zone (Type 1 or Type 2) during colposcopic evaluation, ensuring that the therapeutic light can completely access the borders of the lesion. Conversely, patients presenting with extensive endocervical canal involvement, suspected MIC, or deep glandular invasion are poor candidates for standard surface PDT due to light penetration limits and should proceed directly to standard excisional therapies.

Current protocols involve the topical application of 10% to 20% w/w 5-ALA or its formulation equivalents (such as specialized HAL hydrogels) directly onto the cervix using a dedicated cervical cap or specialized drug-delivery matrix. Following an optimized incubation period of 3 to 4 hours to permit maximum intracellular PpIX accumulation, the cervical tissue is exposed to a red light source (typically 635 nm via semiconductor lasers or specialized LED arrays) delivering an ectocervical fluence of 100–150 J/cm² and an endocervical dose of up to 300 J/cm² via cylindrical diffuser fibers. The procedure is performed in an outpatient setting, requires minimal or no local anesthesia, and features high patient adherence. Mild localized side effects, including transient pelvic cramping, low-grade vaginal discharge, and focal erythema, are self-limiting and resolve within 72 hours.

Incorporating PDT into routine clinical pathways has gained significant momentum; for instance, the Photocure’s photodynamic products officially support the use of non-invasive, device-integrated photodynamic systems as a viable alternative to passive surveillance or immediate surgical excision in young, compliant cohorts, setting a precedent for broader international guideline adaptation.

### 6.3 Emerging clinical biomarkers for treatment stratification

To transition from empirical application to precision oncology, cervical PDT must leverage objective molecular biomarkers to predict individual therapeutic response. Beyond monitoring post-treatment cytological regression and tissue normalization via traditional p16/Ki-67 immunohistochemical staining, genotype-specific hrHPV viral load tracking represents an accessible clinical predictive tool. Patients presenting with high baseline viral loads composed primarily of non-HPV16/18 genotypes frequently exhibit accelerated virological clearance compared to individuals harboring highly integrated, metabolically aggressive HPV16 or HPV18 strains.

At the metabolic level, the baseline selectivity and ultimate efficacy of 5-ALA-PDT are dictated by a complex, tumor-specific dysregulation of intracellular transport systems, heme biosynthetic enzymes, and mitochondrial iron-handling proteins. The gene differential profile of crucial proteins for heme biosynthesis may dictate the selective PpIX production upon 5-ALA PDT in tumor cells. For example, the selective down-regulation of *FECH* and mitochondrial iron transporters (*MRFN1* and *MRFN2*) in tumor populations compromises mitochondrial iron metabolism, creating an enzymatic bottleneck that limits the conversion of PpIX into heme (63). As a result, tumor cells exhibit an intrinsic propensity for hyper-accumulating PpIX, rendering them uniquely vulnerable to PDT-mediated phototoxicity compared to healthy surrounding architecture (63, 122). Increased expression of either ABCB6 or biosynthetic enzymes (e.g., CPOX) enhances also ALA-induced PpIX accumulation and photodamage (121, 123, 124). Conversely, overexpression of *FECH, ABCG2* and*, MRFN2* reduces PpIX accumulation (118, 124). Consequently, cellular transporters (ABCB6, ABCG2), mitochondrial iron regulators (MRFN1, MRFN2), and biosynthetic enzymes (FECH, CPOX) collectively modulate the intracellular porphyrin environment, serving as vital predictive biomarkers for 5-ALA-PDT efficacy and safety in cervical lesions.

Recognizing mitochondrial iron-metabolization and the heme biosynthetic cascade as key regulatory checkpoints introduces novel diagnostic and therapeutic opportunities. For instance, the adjuvant clinical administration of iron chelators, such as DFO, can effectively bypass metabolic bottlenecks by inhibiting FECH activity through substrate depletion, significantly amplifying intracellular PpIX accumulation and light-induced cytotoxicity within resistant tumor cells (122). Similarly, the targeted modulation of heme HO1 and HO2 expression has emerged as a viable approach to suppress downstream heme turnover and preserve high transient photosensitizing pools (124). Systematically mapping these genetic and metabolic profiles provides an objective framework to predict patient responses, ultimately supporting the clinical implementation of 5-ALA in both targeted photodynamic eradication and advanced, fluorescence-guided oncologic procedures (125).

### 6.4 Translational challenges, economic viability, and future horizons

Despite clear clinical advantages, several structural barriers prevent the universal adoption of PDT in global gynecologic practice. The most significant obstacles include the lack of fully unified international guidelines, the substantial capital expenditure required to acquire medical laser systems, and the intensive training required for colposcopists to transition from subjective visual assessment to strict molecular and histological endpoints. Furthermore, the multi-hour outpatient incubation requirement imposes an operational constraint on busy clinical departments.

To circumvent these operational bottlenecks, recent biomedical engineering breakthroughs have introduced fully integrated, portable, and disposable cervical photodynamic delivery systems. For instance, Cevira® (APL-1702), a hexyl aminolevulinate (HAL)-based photodynamic therapy for HSIL, combines a specialized HAL ointment with a miniaturized intravaginal cold-light device (NCT04484415). Underscoring its global advancement, Cevira^®^ achieved commercial approval from China’s NMPA in March 2026, concurrent with an active European Medicines Agency (EMA) review and an agreed U.S. FDA Phase III trial design. This innovation enables brief outpatient placement, allowing the patient to complete the illumination phase at home while maintaining normal daily activities, radically improving patient convenience and institutional throughput. However, it requires investment in both consumable products (the drug/ointment) and capital equipment (such as miniaturized intravaginal light devices).

From an economic standpoint, formal cost-effectiveness analyses comparing PDT to traditional excisional methods reveal a favorable long-term financial profile. While the initial direct medical costs of photodynamic PSs and specialized light delivery systems exceed the immediate cost of a standard LEEP or cold knife conization, PDT offers advantages in safety and fertility preservation that offset these upfront expenses. For example, PDT prevents major obstetric complications, such as 1) premature births requiring prolonged Neonatal Intensive Care Unit (NICU) admissions; 2) cervical stenosis requiring surgical dilation; and 3) late-term miscarriages. When factoring in its repeatability without tissue loss and its superior performance in preventing recurrent disease through localized immune activation, PDT represents a highly cost-effective intervention in modern gynecologic oncology.

## 7 Future perspectives

Given that 5-ALA is a precursor in the heme biosynthetic pathway, systemic iron metabolism significantly impacts the accumulation of the active photosensitizer PpIX. Future clinical studies should integrate standardized biochemical assays -including serum iron, ferritin, and transferrin saturation, alongside hepcidin measurements to elucidate the impact of iron regulation on PDT efficacy. Hepcidin measurement, increasingly available through validated ELISA platforms, could provide additional insight into systemic iron regulation, since it plays a crucial role in controlling iron absorption, storage, and release (140). Specialized assays for FECH activity, FRTX, and HO-1/HO-2 could further elucidate mitochondrial iron handling and heme turnover. Together, these clinically accessible biomarkers offer a comprehensive profile of iron status and their impact on PpIX accumulation, thereby enabling rigorous assessment of PDT efficacy.

Additionally, the evaluation of autophagic biomarkers (LC3-II and p62) and proliferative markers (Ki-67, p16) will be essential for monitoring therapeutic responses. Expanding these investigations to encompass a broader range of hrHPV genotypes (e.g., HPV31, 33, 35, 39, 45, 51, 52, 56, 58, 59, 66, and 68) and extending follow-up periods to 24 months will be vital to validating the long-term durability and obstetric safety of 5-ALA-PDT as a gold-standard approach, particularly regarding fertility preservation and obstetric outcome (135).

## 8 Concluding remarks

The primary take-home message for contemporary gynecological practice is the necessary transition from a purely excisional paradigm to one of metabolic precision. While traditional procedures like the LEEP remain effective at removing dysplastic tissue, they impose a structural and physiological burden on the cervix that often translates to adverse obstetric outcomes, including cervical insufficiency and preterm birth. As we discussed here, clinical data now confirms that photodynamic therapy, specifically using 5-aminolevulinic acid, achieves lesion remission rates between 90-97%, matching the efficacy of surgery while preserving the anatomical integrity of the cervical stroma. This makes PDT not merely a “conservative” option, but a targeted oncological intervention with the goal of fertility preservation. PDT may preserve cervical architecture, leading to uncomplicated term pregnancies.

Looking toward future perspectives, the success of PDT will be defined by “molecular triage” rather than visual inspection alone. The identification of biological determinants of resistance, such as the overexpression of the ABCG2 efflux transporter or high baseline FECH activity, will allow clinicians to personalize treatment protocols. These protocols could adjust photosensitizer incubation times or utilize potent ester derivatives like HAL to overcome metabolic bottlenecks. Furthermore, the integration of autophagy markers offers a new window into the cellular stress response, helping to distinguish between a successful therapeutic “autophagic block” and protective survival mechanisms that could lead to recurrence.

Finally, for PDT to be solidified within international guidelines, rigorous diagnostic endpoints must replace the subjectivity of colposcopic normalization. Evidence indicates that visual assessment alone can miss up to one-third of persistent high-grade disease, meaning that future protocols must mandate histological verification and genotype-specific hrHPV clearance as the definitive criteria for cure. By combining this diagnostic rigor with standardized biophysical parameters—maintaining irradiances near 100 mW/cm² and 100-120 J/cm², PDT can be established as a precision-based standard of care that safeguards both the oncological safety and the reproductive future of women with HSIL.

## Glossary

4-HNE: 4-Hydroxy-2-Nonenal
4E-BP1: Eukaryotic Translation Initiation Factor 4E-Binding Protein 1
5-ALA: 5-Aminolevulinic Acid
ABCB6: ATP-Binding Cassette Subfamily B Member 6
ABCG2: ATP-Binding Cassette Subfamily G Member 2
ALAD: Aminolevulinic Acid Dehydratase
AMPK: Adenosine Monophosphate-Activated Protein Kinase
ANT: Adenine Nucleotide Translocator
APAF-1: Apoptotic Peptidase Activating Factor 1
APC: Antigen-Presenting Cell
ATF6: Activating Transcription Factor 6
ATG: Autophagy-Related
ATP: Adenosine Triphosphate
BAX/BAK: Bcl-2-Associated X Protein/Bcl-2 Homologous Antagonist/Killer
BIM: Bcl-2-Like Protein 11
BTLA: B and T Lymphocyte Attenuator
BVR: Biliverdin Reductase
CAD: Caspase-Activated DNase
CaMKKβ: Calcium/Calmodulin-Dependent Protein Kinase Kinase 2
CC: Cervical Cancer
CCSCs: Cervical Cancer Stem Cells
CD: Cluster of Differentiation
CHOP: C/EBP Homologous Protein
CIN: Cervical Intraepithelial Neoplasia
CL: Cardiolipin
CLEAR: Coordinated Lysosomal Expression and Regulation
CO: Carbon Monoxide
CPO III: Coproporphyrinogen III
CPOX: Coproporphyrinogen III Oxidase
CQ: Chloroquine
CR: Complete Response
CRT: Calreticulin
CSS: Cervical Stem Cell-Related Gene Signatures
CTLA4: Cytotoxic T-Lymphocyte Associated Protein 4
Cyp-D: Cyclophilin D
Cyt c: Cytochrome c
DAMPs: Damage-Associated Molecular Patterns
DCCCs: Dormant Cervical Cancer Cells
DCs: Dendritic Cells
DMT1: Divalent Metal Transporter 1
DNA: Deoxyribonucleic acid
EGFR: Epidermal Growth Factor Receptor
ELISA: Enzyme-Linked Immunosorbent Assay
EMT: Epithelial-Mesenchymal Transition
ER: Endoplasmic Reticulum
ERK: Extracellular Signal-Regulated Kinase
ERp57: Endoplasmic Reticulum Resident Protein 57
ERp72: Endoplasmic Reticulum Resident Protein 72
ETC: Electron Transport Chain
FECH: Ferrochelatase
FRTX: Frataxin
GPX4: Glutathione Peroxidase 4
GSDMD: Gasdermin D
HAL: Hexyl Aminolevulinate
HCQ: Hydroxychloroquine
HMB: Hydroxymethylbilane
HMGB1: High Mobility Group Box 1
HO-1/HO-2: Heme Oxygenase 1/2
HPV: Human Papillomavirus
hrHPV: High-risk Human Papillomavirus
HSIL: High-grade Squamous Intraepithelial Lesion
HSP: Heat Shock Protein
ICC: Invasive Cervical Cancer
ICD: Immunogenic Cell Death
IL: Interleukin
IMM: Inner Mitochondrial Membrane
JNK: c-Jun N-Terminal Kinase
KLF4: Kruppel-Like Factor 4
KRT: Keratin
LC3: Microtubule-Associated Protein 1 Light Chain 3
LCR: Long Control Region
LEEP: Loop Electrosurgical Excision Procedure
LLETZ: Large Loop Excision of the Transformation Zone
LSIL: Low-Grade Squamous Intraepithelial Lesion
MAL: Methyl Aminolevulinate
MDA: Malondialdehyde
MEK: Mitogen-Activated Protein Kinase Kinase
MIC: Microinvasive Carcinoma
MLKL: Mixed Line lineage Kinase Domain-Like Pseudokinase
MOMP: Mitochondrial Outer Membrane Permeabilization
mPTP: Mitochondrial Permeability Transition Pore
MRFN: Mitoferrin
mtDNA: Mitochondrial Deoxyribonucleic acid
mTOR: Mammalian Target of Rapamycin
mTORC1: Mammalian Target of Rapamycin Complex 1
mtROS: Mitochondrial Reactive Oxygen Species
NADPH: Nicotinamide Adenine Dinucleotide Phosphate
NANOG: NANOG Homeobox
NLRP3: NLR Family Pyrin Domain Containing 3
OCT4: POU Class 5 Homeobox 1
OMM: Outer Mitochondrial Membrane
ORF: Open Reading Frame
OXPHOS: Oxidative Phosphorylation
p16: Cyclin-Dependent Kinase Inhibitor 2A
p53: Tumor Protein p53
p62: Sequestosome 1/Ubiquitin-Binding Protein p62
p63: Tumor Protein p63
PARP-1: Poly(ADP-Ribose) Polymerase 1
PAX1/PAX1hm: Paired Box 1/PAX1 Hypermethylation
PBGD: Porphobilinogen Deaminase
PD-1: Programmed Cell Death 1
PDT: Photodynamic Therapy
PE: Phosphatidylethanolamine
PEPT1/PEPT2: Peptide Transporter 1/2
PERK: Eukaryotic Translation Initiation Factor 2 Alpha Kinase 3
PBG: Porphobilinogen
PPGIX: Protoporphyrinogen IX
PpIX: Protoporphyrin IX
PPOX: Protoporphyrinogen Oxidase
pRb: Retinoblastoma Protein
PS: Photosensitizer
PUFA/PUFA-OOH: Polyunsaturated Fatty Acid/Polyunsaturated Fatty Acid Hydroperoxide
RAF: Raf-1 Proto-Oncogene, Serine/Threonine Kinase
RAS: Ras Oncogene Family
Rheb: Ras Homolog, mTORC1 Binding
RIPK1/RIPK3: Receptor-Interacting Serine/threonine Kinase 1/3
RNA: Ribonucleic Acid
ROS: Reactive Oxygen Species
S6K/S6K1: Ribosomal Protein S6 Kinase B1
SCJ: Squamocolumnar Junction
SNAI2: Snail Family Transcriptional Repressor 2
SOX2: SRY-Box Transcription Factor 2
TAZ: WW Domain Containing Transcription Regulator 1
TCT: ThinPrep Cytology Test
TFEB: Transcription Factor EB
TFRC: Transferrin Receptor
TIGIT: T-Cell Immunoreceptor with Ig and ITIM Domains
TNF-α: Tumor Necrosis Factor Alpha
TSC: Tuberous Sclerosis Complex
TZ: Transformation Zone
ULK1: Unc-51 Like Autophagy Activating Kinase 1
UPR: Unfolded Protein Response
UROD: Uroporphyrinogen Decarboxylase
UROS: Uroporphyrinogen III Synthase
URR: Upstream Regulatory Region
WHO: World Health Organization
YAP: YAP1, Transcriptional Coactivator
