# Supplementary material for: Photodynamic therapy: a promising alternative for high-grade squamous intraepithelial lesion treatment
Source: Front Oncol. 2026 Aug 7;16:1899399. doi: 10.3389/fonc.2026.1899399 (PMC13494708; doi:10.3389/fonc.2026.1899399)
Supplement: Supplementary file 1 [file Table1.docx]

| Synonyms | **5-aminolevunic acid (5-ALA)** | **Methyl aminolevulinate (MAL)** | **Hexyl aminolevulinate (HAL)** |
| --- | --- | --- | --- |
|  | 5-ALA, 5-Aminolevulinic acid, 5-aminolevulinate, δ-aminolevulinate, delta-aminolevulinic acid, δ-aminolevulinic acid, | Methyl 5-aminolevulinate, aminolevulinic acid methyl ester, methyl delta-aminolevulinate, methyl δ-aminolevulinate | 5-Aminolevulinic acid hexyl ester, hexyl-aminolevulinate, ALA hexyl ester,  hexa ALA, hexylaminolevulinate |
| Cellular locations | Mitochondria | Membrane | Not available |
| **Biospecimen Locations** | Blood (0.35 +/- 0.09 µM)  Urine (1.28 +/- 0.57 µmol/mMol creatinine) | Blood  Urine | Not available |
| Structure |  |  |  |
| State | Solid | Solid | Solid |
| Chemical formula | C_5_H_9_NO_3_ | C_6_H_11_NO_3_ | C_11_H_21_NO_3_ |
| Molecular Weight | 131.13 g/mol | 145.16 g/mol | 215.29 g/mol |
| Water solubility | 173.0 mg/mL  1000 mg/mL at 25 °C | 220 mg/mL | 0.511 mg/mL |
| LogP | -1.5 | -1.2 | 1.84 |
| pKa | 4.05  7.84 (strongest basic) | 7.83 (strongest basic) | 7.83 (strongest basic) |
| Physiological charge | 0 | 1 | 1 |
| Melting point | 156-158 °C | Not available | Not available |
| Absorption | Oral bioavailability is 50-60% | Topically absorbed via skin stratum corneum | Absolute bioavailability 7% |
| Biological  half-life | Topical dosing: ~5.7 hours;  Oral dosing: < 1 hour | Tissue-specific conversion to PpIX occurs within hours | Biphasic: initial half-life of 39 min;  terminal half-life of ~76 hours |
| Routes | Oral, topical | Topical | Intravesical |
| Dosage forms | Gel (78 – 100 mg/g); Patch (8 mg); Plaster (8 mg); Powder for oral solution (30 mg/mL); Powder for topical solution (20% w/w) | Cream (168 mg/g) | Powder for intravesical solution (85 – 100mg) |
| Product ingredient | Aminolevulinic acid hydrochloride | Methyl aminolevulinate hydrochloride | Hexaminolevulinate hydrochloride |
| Optimal concentration for topical usea | 5 - 20% w/w (0.3 - 1.2 M)^a^ | 20% w/w (1.1 M)^a^ | 5 - 40% w/w (0.2 -1.6 M)^a^ |
| Products | Ameluz®, Gleolan®, Gliolan®  Levulan Kerastik® | Metvix®, Metvixia® | Cysview®, Hexvix®, Cevira® |
| Approved Clinical Indication | Actinic Keratosis (Topical)  Glioma visualization (Oral) | Actinic Keratosis  Basal Cell Carcinoma (Topical) | Bladder Cancer Detection (Intravesical instillation) |
| Price | Levulan Kerastick® / Ameluz®:  $350 – $600 per unit (Institutional/WAC)  Gleolan®: $2,000+ per oral vial | Metvixia® 16.8% cream:  $150 – $300 per 2g tube (WAC market value) | Cysview® / Hexvix® 100 mg kit:  $900 – $1,300 per instillation kit |
| Induced PpIX Photophysical properties | 405 nm (Soret band excitation);  600 to 635 nm (Q-bands) | 405 nm (Soret band excitation);  570 to 670 nm (Q-bands) | 405 nm (Soret band excitation);  360 to 450 nm (UV-blue absorption diagnostic range) |
| Optimal light activation wavelength | 635 nm (Red light for PDT)  405 nm (Blue light for fluorescence diagnosis) | 635 nm (Red light for PDT) | 635 nm (Red light for PDT)  405 nm (Blue light for fluorescence diagnosis) |
| Fluence | 80–140 J/cm^2^ | 100–180 J/cm^2^ | 50–100 J/cm^2^ |
| Irradiance | 80–200 mW/cm^2^ | 80–100 mW/cm^2^ | 6 mW/cm^2^ |
| DrugBank number | [DB00855](https://go.drugbank.com/drugs/DB00855) | [DB00992](https://go.drugbank.com/drugs/DB00992) | [DB06261](https://go.drugbank.com/drugs/DB06261) |
| PubChem CID | [137](https://pubchem.ncbi.nlm.nih.gov/compound/137) | [157922](https://pubchem.ncbi.nlm.nih.gov/compound/157922) | [6433083](https://pubchem.ncbi.nlm.nih.gov/compound/6433083) |
| Human Metabolome Database (HMDB) | [HMDB0001149](https://www.hmdb.ca/metabolites/HMDB0001149) | [HMDB0015127](https://www.hmdb.ca/metabolites/HMDB0015127) | Not available |

a: assuming the solution density is 1.0 g/mL.

The data was extracted from previous work (1,2,11,3–10) and databases.

References

1.	Fu Y, Bao Y, Hui Y, Gao X, Yang M, Chang J. Topical photodynamic therapy with 5-aminolevulinic acid for cervical high-risk HPV infection. *Photodiagnosis Photodyn Ther* (2016) 13:29–33. doi: 10.1016/j.pdpdt.2015.12.004

2.	Uehlinger P, Zellweger M, Wagnières G, Juillerat-Jeanneret L, Van Den Bergh H, Lange N. 5-Aminolevulinic acid and its derivatives: Physical chemical properties and protoporphyrin IX formation in cultured cells. *J Photochem Photobiol B Biol* (2000) 54:72–80. doi: 10.1016/S1011-1344(99)00159-1

3.	Chen F, Hillemanns P, Ruan H, Chen X, Wang Y, Novak Z, Zhang Y, You Z, Wei B, Lu W, et al. Photodynamic therapy for high-grade squamous intraepithelial lesions: A randomized controlled trial. *Med* (2025) 6: doi: 10.1016/j.medj.2025.100851

4.	Cai H, Che Y, Sun X, Yu X, Yu F, Hou F, Wang Y, Ma T. Clinical outcome of photodynamic therapy for the treatment of cervical intraepithelial neoplasia grade 2. *Photodiagnosis Photodyn Ther* (2022) 39:102906. doi: 10.1016/j.pdpdt.2022.102906

5.	Han Q, Guo H, Wu Z, Shi J, Zhang X. Efficacy and Safety of 5-Aminolevulinic Acid Photodynamic Therapy for Treating Cervical and Vaginal Intraepithelial Neoplasia. *Pharmaceutics* (2024) 16: doi: 10.3390/pharmaceutics16050627

6.	Hillemanns P, Garcia F, Petry KU, Dvorak V, Sadovsky O, Iversen OE, Einstein MH. A randomized study of hexaminolevulinate photodynamic therapy in patients with cervical intraepithelial neoplasia 1/2. *Am J Obstet Gynecol* (2015) 212:465.e1-465.e7. doi: 10.1016/j.ajog.2014.10.1107

7.	Ma L, Gao X, Geng L, You K, Wu Z, Li Y, Han Q, Wang Y, Guo H. Efficacy and safety of photodynamic therapy mediated by 5-aminolevulinic acid for the treatment of cervical intraepithelial neoplasia 2: A single-center, prospective, cohort study. *Photodiagnosis Photodyn Ther* (2021) 36:102472. doi: 10.1016/j.pdpdt.2021.102472

8.	Tang Y, Su Y, Xu Y, Zhang Y, Shen Y, Qin L, Zhang L, Cao L, Zhou Y, Zhang T, et al. Therapeutic effects of topical photodynamic therapy with 5-aminolevulinic acid on cervical high-grade squamous intraepithelial lesions. *Photodiagnosis Photodyn Ther* (2022) 39:102884. doi: 10.1016/j.pdpdt.2022.102884

9.	Wang L, Liu X, Zhang J, Song M, Liu H, Xu Y, Meng L, Zhang Y, Jia L. Comparison of 5-ALA-PDT and LEEP of cervical squamous intraepithelial neoplasia (CIN2) with high-risk human papillomavirus infection in childbearing age women: A non-randomized controlled pilot study. *Photodiagnosis Photodyn Ther* (2024) 46:104044. doi: 10.1016/j.pdpdt.2024.104044

10.	Wei Y, Gu L, Zhang Y, Yang Q, Chen F, Hong Z, Di W, Qiu L. Efficacy of ALA-PDT in treating cervical low-grade squamous intraepithelial lesions with high-risk HPV patients: A multicentre randomized controlled trial. *Int J Cancer* (2025) 157:908–915. doi: 10.1002/ijc.35450

11.	Zhang Y, Su Y, Tang Y, Qin L, Shen Y, Wang B, Zhou Y, Zhang M, Zhang T. Management of patients with positive margin after conization for high-grade cervical intraepithelial lesions. *Lasers Surg Med* (2022) 54:1099–1106. doi: 10.1002/lsm.23585
